# An interchange property for the rooted phylogenetic subnet diversity on phylogenetic networks

**DOI:** 10.1007/s00285-024-02142-4

**Published:** 2024-10-04

**Authors:** Tomás M. Coronado, Gabriel Riera, Francesc Rosselló

**Affiliations:** 1 Department of Mathematics and Computer Science, University of the Balearic Islands, Palma, E-07122 Spain; 2 Balearic Islands Health Research Institute (IdISBa), Palma, E-07010 Spain

**Keywords:** Phylogenetic network, Level-*k* network, Phylogenetic subnet diversity, Phylogenetic subnet diversity optimization problem, 92B10

## Abstract

Faith’s Phylogenetic Diversity (PD) on rooted phylogenetic trees satisfies the so-called strong exchange property that guarantees that, for every two sets of leaves of different cardinalities, a leaf can always be moved from the larger set to the smaller set in such a way that the sum of the PD values does not decrease. This strong exchange property entails a simple polynomial-time greedy solution to the PD optimization problem on rooted phylogenetic trees. In this paper we obtain an exchange property for the rooted Phylogenetic Subnet Diversity (rPSD) on rooted phylogenetic networks, which involves a more complicated exchange of leaves. We derive from it a polynomial-time greedy solution to the rPSD optimization problem on rooted semibinary level-2 phylogenetic networks.

**Supplementary Information:**

The online version contains supplementary material available at 10.1007/s00285-024-02142-4.

## Introduction

Over the last few centuries, human activity has caused the destruction of natural habitats at an unprecedented pace, resulting in a major episode of biodiversity extinction (Kolbert 2014). Urgent action is required to combat extinction and preserve biodiversity, but there are challenges, including a lack of funding and uncertainties about conservation strategies. Consequently, there has been an increasing need to provide criteria for defining priorities and proposing variables that allow quantification of biodiversity.

The traditional approach to assessing biodiversity based on species counts, species richness, and number of endemic species has limitations. For instance, this type of data is so heterogeneous that it can be difficult to compare across different sites and times (Gaston 1996). The approach based on lists of threatened species also has its drawbacks: for example, changes in the composition of these lists may represent changes in knowledge of species status rather than changes in the status itself (Possingham et al. 2002). Finally, measures of biodiversity based solely on species have been criticized for treating all species as equal, without regard to their functional roles in the ecosystem or their evolutionary history (Faith 1992).

A feature of species that may influence their biodiversity value is their evolutionary distinctness. A species with few close living evolutionary relatives is considered more worthy of protection than a species with many close genetically and phenotypically similar relatives (McNeely et al. 1990). At the beginning of the 1990s, the qualitative value afforded to evolutionarily distinct species was replaced by quantitative measures of phylogenetic distinctness. One of the first published measures of biodiversity based on phylogenetic information was Faith’s *phylogenetic diversity*, PD (Faith 1992). The PD value of a set of species placed in the leaves of a phylogenetic tree is defined as the total weight (i.e., the sum of the branch lengths) of the spanning tree connecting the root and these leaves. In its original formulation, the branch lengths represented the number of changes in phenotypic characters, and PD measured the diversity of phenotypic characters in a set of species. In the current usual interpretation of phylogenetic trees, branch lengths represent evolutionary time, which is assumed to be positively correlated with character variation.

Since its introduction, PD has been widely studied and applied (Pellens and Grandcolas 2016). One of its most useful properties, both from the formal and the applicability point of view, is the possibility of efficiently finding and characterizing all subsets of species in a phylogenetic tree of a given size with maximal PD value by means of a very simple greedy algorithm (Pardi and Goldman 2005; Steel 2005); for instance, for a recent application to the analysis of SARS-CoV-2 phylogeny, see Zhukova et al. (2021). The basis of this result is the so-called *strong exchange property* stating that for every pair of sets of leaves $X,X'$ with $|X|>|X'|$, we can always move a leaf from *X* to $X'$ without decreasing the sum of the PD values.

Faith’s PD is defined on evolutionary histories modelled by means of phylogenetic trees. But phylogenetic trees can only cope with speciation events due to mutations, where each species other than the universal common ancestor has only one parent in the evolutionary history (its parent in the tree). It is clearly understood now that other speciation events, which cannot be properly represented by means of single arcs in a tree, play an important role in evolution (Doolittle 1999). These are *reticulate events*, like genetic recombinations, hybridizations, or lateral gene transfers, where a species is the result of the interaction between several parent species. This has lead to the introduction of *phylogenetic networks* as models of phylogenetic histories that allow to include these reticulate events (Huson et al. 2010). Faith’s PD has been extended to split networks^1^(Spillner et al. 2008) and to rooted phylogenetic networks (Wicke and Fischer 2018; Bordewich et al. 2022); as a matter of fact, several generalizations to rooted phylogenetic networks have been proposed, the most natural of which is the *rooted Phylogenetic Subnet Diversity*, rPSD, introduced by Wicke and Fischer (2018) and renamed *AllPaths-PD* by Bordewich et al. (2022).

It has been proved that the PD optimization problem can be solved efficiently on *circular* split networks^2^ using integer programming (Chernomor et al. 2016; Spillner et al. 2008), as well as (for rPSD) on the simplest class of non-tree rooted phylogenetic networks, the so-called *galled trees*, by reducing it to sets of linear size of minimum-cost flow problems (Bordewich et al. 2009, 2022). It is also known that these optimization problems are in general NP-hard on rooted phylogenetic networks (Bordewich et al. 2022) and on split networks (Chernomor et al. 2016).

In this paper we focus on the extension of the greedy optimization algorithm for PD on phylogenetic trees to rPSD on rooted phylogenetic networks. As we have mentioned, the greedy algorithm on phylogenetic trees is a consequence of the strong exchange property for PD that guarantees that, given two sets of leaves of different cardinalities, we can always move some element from the larger set to the smaller one without lowering the sum of the PD values. It is easy to check that this strong exchange property for rPSD is no longer valid even on galled trees (Bordewich et al. 2022). So, our first main contribution is its generalization to rPSD through a more involved exchange of leaves than simply moving one leaf from one set to another.

Our exchange property then allows us to strengthen the result of Bordewich et al. on galled trees, by proving that every rPSD-optimal set of *m* leaves in a galled tree is always obtained from an rPSD-optimal set of $m-1$ leaves by either optimally adding a leaf or optimally replacing a leaf by a pair of leaves. It also allows us to give polynomial time greedy solutions for the rPSD problem on semibinary level-2 networks and semi-3-ary level-1 networks, the next complexity level of rooted phylogenetic networks (see §2.1 for the definitions). On the negative side, we have not been able to deduce from it a greedy algorithm for semibinary level-3 or semi-4-ary level-1 networks and the problem for these more general classes remains open.

This paper is organized as follows. In Sect. 2.1 we define the concepts necessary to understand this work, including a generalization of the Phylogenetic Diversity due to Wicke and Fischer (2018), together with its properties and an example. Section 3 contains the main result of this manuscript, Theorem 1, and Sect. 4 exposes some of its applications to galled trees and to semi-*d*-ary level-*k* networks, for particular instances of *d* and *k*. We end in Sect. 5 with some concluding remarks. The proof of Theorem 1 together with two required lemmas can be found in the Appendix and proofs of additional results can be found in the Supplementary Material.

## Preliminaries

### Phylogenetic networks

Let $\Sigma$ be a finite set of labels. By a *phylogenetic network* on $\Sigma$ we understand a rooted directed acyclic simple graph where each node of in-degree $\geqslant 2$ has out-degree exactly 1 and whose *leaves* (i.e., its nodes of out-degree 0) are bijectively labeled by $\Sigma$ (Huson et al. 2010). A *phylogenetic tree* is simply a phylogenetic network without nodes of in-degree $\geqslant 2$. Let us point out here that, although the usual definition of phylogenetic tree and network forbids, for reconstructibility reasons, the existence of *elementary nodes*, that is, of nodes of in-degree $\leqslant 1$ and out-degree 1, we shall allow their existence in order to simplify some statements and proofs.

Let *N* be a phylogenetic network. We shall denote its *root* (i.e., its only node of in-degree 0) by *r* and its sets of nodes and arcs by *V*(*N*) and *E*(*N*), respectively, and we shall always identify its leaves with their corresponding labels. Given two nodes *u*, *v* in *N*, we say that *v* is a *child* of *u*, and also that *u* is a *parent* of *v*, when $(u,v)\in E(N)$. A node in *N* is of *tree type*, or a *tree node*, when its in-degree is $\leqslant 1$, and a *reticulation* when its in-degree is $\geqslant 2$ (and hence, its out-degree is 1). We shall say that *N* is *semi*-*d*-*ary* when all its reticulations have in-degree $\leqslant d$, and that *N* is *binary* when it is semibinary and all its internal tree nodes have out-degree 2.

**Fig. 1:**
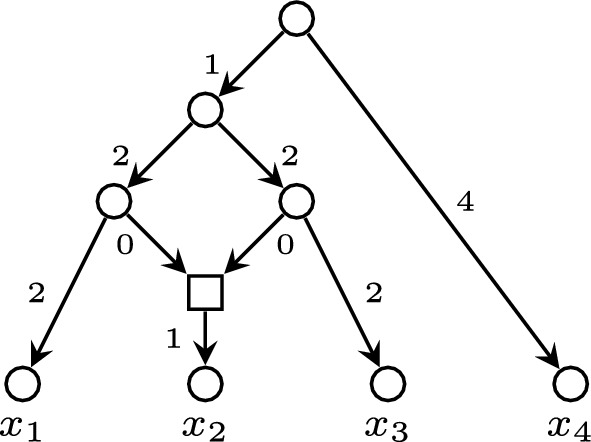
A weighted phylogenetic network. The tree nodes are represented by circles, the reticulation by a square, and the arcs’ labels represent their weights

We shall denote a (directed) path in *N* from a node *u* to a node *v* by $u\!\rightsquigarrow \! v$. The *intermediate* nodes of a path $u\!\rightsquigarrow \! v$ are the nodes involved in it other than *u* and *v*. For every $u,v\in V(N)$, we say that *v* is a *descendant* of *u*, and also that *u* is an *ancestor* of *v*, when there exists a path $u\!\rightsquigarrow \! v$, and that *v* is a *descendant* of an arc $e=(u',u)$ when it is a descendant of its end *u*. In particular, every node is an ancestor, and a descendant, of itself. If *v* is a descendant of *u* and $u\ne v$, we shall say that it is a *proper descendant* of *u*. A set of nodes $V_0\subseteq V(N)$ is *independent* when no node in it is a proper descendant of any other node in it.

For every $v\in V(N)$, its *cluster* $C_N(v)\subseteq \Sigma$ (or simply *C*(*v*) when *N* is clear from the context), is the set of (labels of) the descendant leaves of *v*, and the *subnetwork of* *N* *rooted at* *v* is the subgraph $N_v$ of *N* induced by the set of all descendants of *v*. $N_v$ is a phylogenetic network on *C*(*v*) with root *v*.

For every $X\subseteq V(N)$, we shall denote the set of all nodes in *N* that are ancestors of nodes in *X* by ${\uparrow }X$. Given an arc $e=(u,u')\in E(N)$, we shall make the abuse of notation of writing $e\in {\uparrow }X$ to mean that *e* has some descendant in *X*, that is, that $u'\in {\uparrow }X$.

A subgraph of a phylogenetic network *N* is *biconnected* when it is connected (as an undirected graph) and it remains connected after removing any node from it together with all arcs incident to this node. Every node and every arc in *N* are biconnected subgraphs. A *biconnected component* of *N* is a maximal biconnected subgraph, and we shall call a biconnected component with more than 2 nodes a *blob*. Every blob ${\mathcal {B}}$ has one, and only one, node that is an ancestor of all its nodes; we call it its *split node*. Every node in a blob ${\mathcal {B}}$ with no child inside ${\mathcal {B}}$ is a reticulation (should it be of tree type, removing its parent would disconnect ${\mathcal {B}}$); we call such reticulations the *exit reticulations* of ${\mathcal {B}}$, and the rest of its reticulations, *internal*. Every node in ${\mathcal {B}}$ has some descendant exit reticulation.

A phylogenetic network is *level*-*k* (Jansson and Sung 2006) when every biconnected component contains at most *k* reticulations. Thus, a level-0 network is a phylogenetic tree. A semibinary level-1 network is also called a *galled tree* (Gusfield et al. 2004); the phylogenetic network in Fig. 1 is a galled tree.

A phylogenetic network *N* is *weighted* when it is endowed with a weight mapping $w:E(N) \rightarrow {\mathbb {R}}_{\geqslant 0}$. The *total weight* of a subgraph of a weighted phylogenetic network is the sum of the weights of all arcs in the subgraph. In particular, the weight of a path is the sum of the weights of its arcs. All phylogenetic networks (and trees) appearing from now on in this paper are assumed to be weighted, usually without any further notice.

### The rooted phylogenetic diversity on phylogenetic trees

Given a finite set $\Sigma$, we shall denote henceforth its set of subsets by $\mathcal {P}(\Sigma )$ and, for every $k\geqslant 0$, the set of all its subsets of cardinality *k* by $\mathcal {P}_k(\Sigma )$.

Given a weighted phylogenetic tree *T* on $\Sigma$, Faith’s *rooted Phylogenetic Diversity* (Faith 1992) is the set function $\textrm{PD}_T:\mathcal {P}(\Sigma )\rightarrow {\mathbb {R}}_{\geqslant 0}$ sending each $X\subseteq \Sigma$ to the total weight of the subtree induced by the ancestors of nodes in *X*:

$$\begin{aligned} \textrm{PD}_T(X)=\sum _{e\in {\uparrow }X} w(e). \end{aligned}$$

This function $\textrm{PD}_T$ on phylogenetic trees satisfies the following *strong exchange property*, introduced by Steel (2005) for unrooted phylogenetic trees: for every phylogenetic tree *T* on $\Sigma$ and for every $X,X'\subseteq \Sigma$ such that $|X'|<|X|$, there exists some $x\in X{\setminus } X'$ such that

$$\begin{aligned} \textrm{PD}_T(X)+\textrm{PD}_T(X')\leqslant \textrm{PD}_T(X'\cup \{x\})+\textrm{PD}_T(X\setminus \{x\}). \end{aligned}$$

For a proof of this fact in the rooted case, see (Steel 2016, §6.4.1).

This strong exchange property for $\textrm{PD}_T$ is the key ingredient in the proof that the simple Algorithm 1 given below produces, for every $k\geqslant 1$, the family ${\mathcal {M}}_k$ of all $\textrm{PD}_T$-*optimal* subsets of $\Sigma$ of cardinality *k*, that is, of all sets of *k* leaves with maximum $\textrm{PD}_T$ value. For this proof in the unrooted case, see Steel (2005); the proof in the rooted case is similar: cf. §6.4.1 in Steel (2016). In particular, given a phylogenetic tree *T* on $\Sigma$, this algorithm provides a polynomial solution to the problem of finding the maximum $\textrm{PD}_T$ value among all members of ${\mathcal {P}}_k(\Sigma )$, and a member of ${\mathcal {P}}_k(\Sigma )$ reaching this maximum.

**Algorithm 1:**
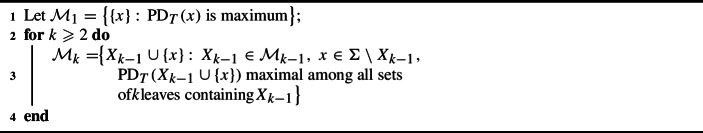
Greedy for phylogenetic trees

### The rooted phylogenetic subnet diversity

Wicke and Fischer (2018) proposed several generalizations of Faith’s rooted Phylogenetic Diversity function to phylogenetic networks. One of them, and possibly the most straightforward, is the *rooted Phylogenetic Subnet Diversity*: the set function $\textrm{rPSD}_N:\mathcal {P}(\Sigma )\rightarrow {\mathbb {R}}_{\geqslant 0}$ sending each $X\subseteq \Sigma$ to the total weight of the subgraph induced by the ancestors of nodes in *X*:

$$\begin{aligned} \textrm{rPSD}_N(X)=\sum _{e\in {\uparrow }X} w(e). \end{aligned}$$

It is clear that if *N* is a phylogenetic tree, then $\textrm{rPSD}_N=\textrm{PD}_N$. When *N* is clear from the context, we shall omit the subscript *N* and simply write $\textrm{rPSD}$.

#### Example 1

On the phylogenetic network *N* depicted in Fig. 1,

$$\begin{aligned} \begin{array}{l} \textrm{rPSD}(x_1)=5,\ \textrm{rPSD}(x_2)=6,\ \textrm{rPSD}(x_3)=5,\ \textrm{rPSD}(x_4)=4,\\ \textrm{rPSD}(\{x_1,x_2\})=8,\ \textrm{rPSD}(\{x_1,x_3\})=9,\ \textrm{rPSD}(\{x_1,x_4\})=9,\\ \textrm{rPSD}(\{x_2,x_3\})=8,\ \textrm{rPSD}(\{x_2,x_4\})=10,\ \textrm{rPSD}(\{x_3,x_4\})=9,\\ \textrm{rPSD}(\{x_1,x_2,x_3\})=10,\ \textrm{rPSD}(\{x_1,x_2,x_4\})=12,\ \textrm{rPSD}(\{x_1,x_3,x_4\})= 13,\\ \textrm{rPSD}(\{x_2,x_3,x_4\})=12,\ \textrm{rPSD}(\{x_1,x_2,x_3,x_4\})=14. \end{array} \end{aligned}$$

For every phylogenetic network *N* on $\Sigma$, $\textrm{rPSD}$ is:

(i) *Monotone nondecreasing*: For every $X\subseteq Y\subseteq \Sigma$, $\textrm{rPSD}(X)\leqslant \textrm{rPSD}(Y)$.
(ii) *Subadditive*: For every $X, Y\subseteq \Sigma$,
  $$\begin{aligned} \textrm{rPSD}(X\cup Y)\leqslant \textrm{rPSD}(X)+\textrm{rPSD}(Y). \end{aligned}$$
(iii) *Submodular*: For every $X\subseteq Y\subseteq \Sigma$ and for every $a\in \Sigma \setminus Y$,
  $$\begin{aligned} \textrm{rPSD}(Y\cup \{a\})-\textrm{rPSD}(Y)\leqslant \textrm{rPSD}(X\cup \{a\})-\textrm{rPSD}(X). \end{aligned}$$

(i) and (ii) are clear. As to (iii), it is proved by Bordewich et al. (2022).

On the negative side, $\textrm{rPSD}$ need not satisfy the strong exchange property, even for the simplest non-tree networks *N*. Indeed, consider again the binary galled tree *N* depicted in Fig. 1. Take $X=\{x_1,x_3,x_4\}$ and $X'=\{x_2,x_4\}$. Then

$$\begin{aligned} \begin{array}{l} \textrm{rPSD}(\{x_1,x_3,x_4\})+\textrm{rPSD}(\{x_2,x_4\}) =23,\\ \textrm{rPSD}(\{x_3,x_4\})+\textrm{rPSD}(\{x_1,x_2,x_4\})= \textrm{rPSD}(\{x_1,x_4\})+\textrm{rPSD}(\{x_2,x_3,x_4\})=21. \end{array} \end{aligned}$$

Therefore, there is no $x\in X\setminus X'$ such that

$$\begin{aligned} \textrm{rPSD}(X)+\textrm{rPSD}(X')\leqslant \textrm{rPSD}(X\setminus \{x\})+\textrm{rPSD}(X'\cup \{x\}). \end{aligned}$$

As a consequence, an $\textrm{rPSD}$-optimal set of cardinality *k* of a phylogenetic network *N* need not contain any $\textrm{rPSD}$-optimal set of cardinality $k-1$. Consider again the galled tree depicted in Fig. 1. Its only set of two labels with largest $\textrm{rPSD}$ value is $\{x_2,x_4\}$ and its only set of three labels with largest $\textrm{rPSD}$ value is $\{x_1,x_3,x_4\}$.

So, Algorithm 1 cannot be used to produce $\textrm{rPSD}$-optimal sets of a given cardinality as it stands. Actually, Bordewich et al. (2022) prove that, given a phylogenetic network *N* on $\Sigma$ and an integer *k*, the problem of finding the maximum $\textrm{rPSD}_N$ value on ${\mathcal {P}}_k(\Sigma )$ is NP-hard. On the positive side, these authors also prove that this problem can be solved in polynomial time on binary galled trees.

## A general exchange property

Let $\Sigma$ be a finite set and $W:\mathcal {P}(\Sigma )\rightarrow {\mathbb {R}}_{\geqslant 0}$ a function. Given $X,X'\subseteq \Sigma$ such that $|X'| < |X|$, a *W*-*improving pair* for $X,X'$ is a pair of sets (*A*, *B*), with $A\subseteq X\setminus X'$, $B\subseteq X'{\setminus } X$, and $|B|<|A|$, such that

$$\begin{aligned} W(X)+W(X')\leqslant W((X\setminus A)\cup B)+W((X'\setminus B)\cup A). \end{aligned}$$

To simplify the notation, given $X\subseteq \Sigma$, $S\subseteq X$ and $T\subseteq \Sigma \setminus X$, we shall denote henceforth $(X\setminus S)\cup T$ by $\tau _{S,T}(X)$.

Given a set

$$\begin{aligned} \mathscr {S}\subseteq \big \{(A,B)\in \mathcal {P}(\Sigma )^2:\, A\cap B=\emptyset ,\ |B|<|A|\big \}, \end{aligned}$$

we shall say that $W:\mathcal {P}(\Sigma )\rightarrow {\mathbb {R}}_{\geqslant 0}$ satisfies the *exchange property with respect to* $\mathscr {S}$ when every pair of sets $X,X' \subseteq \Sigma$ with $|X'| < |X|$ has a *W*-improving pair in $\mathscr {S}$. So, Steel’s *strong exchange property* for phylogenetic trees mentioned in §2.2 says that, for every phylogenetic tree *T* on $\Sigma$, $\textrm{PD}_T: \mathcal {P}(\Sigma )\rightarrow {\mathbb {R}}_{\geqslant 0}$ satisfies the exchange property with respect to

$$\begin{aligned} \mathscr {S}_0(\Sigma )=\big \{(\{x\},\emptyset ):\, x\in \Sigma \big \}. \end{aligned}$$

As we have seen, this is no longer true for $\textrm{rPSD}$ on galled trees. The main result in this paper, Theorem 1, says that $\textrm{rPSD}$ satisfies, on every semi-*d*-ary level-*k* phylogenetic network on $\Sigma$, the exchange property with respect to a larger family of pairs of subsets $\mathscr {S}_{k,d}(\Sigma )$ whose description only depends on *k* and *d*. These families are, when $k=1$,

$$\begin{aligned} \mathscr {S}_{1,d}(\Sigma ) =\, \mathscr {S}_{0}(\Sigma ) \cup \big \{(A,B)\in \mathcal {P}(\Sigma )^2:\, A\cap B=\emptyset ,\ 1\leqslant |B|< |A|\leqslant d \big \} \end{aligned}$$

and, when $$k\geqslant 2$$,

$$\begin{aligned} \mathscr {S}_{k,d}(\Sigma )&= \mathscr {S}_{0}(\Sigma )\\&\quad \ \cup \big \{(A,B)\in \mathcal {P}(\Sigma )^2:\, A\cap B=\emptyset ,\ 1\leqslant |B|< |A|< dk,\\&\quad |A|-|B|\leqslant (d-1)k \big \}. \end{aligned}$$

From now on, when it is unnecessary to explicit the set of labels $\Sigma$, we shall omit it from the notation of these families.

Given *k* and *d*, the cardinalities of these families of sets are polynomial in $|\Sigma |=n$: $|\mathscr {S}_0|=n$ and

$$\begin{aligned} | \mathscr {S}_{1,d}|&= n+\sum _{j=2}^d\sum _{i=1}^{j-1} \left( {\begin{array}{c}n\\ j\end{array}}\right) \left( {\begin{array}{c}n-j\\ i\end{array}}\right) ,\\ | \mathscr {S}_{k,d}|&= n+\sum _{j=2}^{dk-1}\sum _{i=j-(d-1)k}^{j-1} \left( {\begin{array}{c}n\\ j\end{array}}\right) \left( {\begin{array}{c}n-j\\ i\end{array}}\right) \text { when}\, k\geqslant 2. \end{aligned}$$

As we announced above, the main result in this section is the following theorem. Since its proof is quite long and technical, in order not to lose the thread of the manuscript we postpone it until Appendix A at the end of the paper.

### Theorem 1

If *N* is a semi-*d*-ary level-*k* phylogenetic network, $\textrm{rPSD}_N$ satisfies the exchange property with respect to $\mathscr {S}_{k,d}$.

The family $\mathscr {S}_{k,d}$ cannot be improved, because there are semi-*d*-ary level-*k* phylogenetic networks *N* and pairs of sets of leaves $X,X'$ with $|X'|<|X|$ having no $\textrm{rPSD}_N$-improving pair (*A*, *B*) with $|A|-|B|<(d-1)k$. The next example describes one such network for $d=2$; it is straightforward to generalize it to the semi-*d*-ary setting for any $d\geqslant 2$

### Example 2

**Fig. 2:**
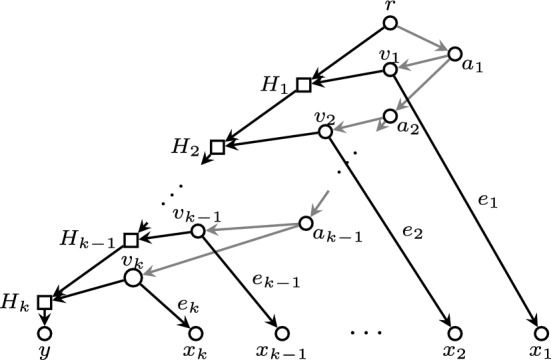
The network *N* in Example 2. The grey arcs form the set $E_0$

Consider the binary level-*k* phylogenetic network *N* on $\Sigma =\big \{y,x_1,\ldots ,x_k\big \}$ depicted in Fig. 2. Assume that all its arcs *e* have weight $w(e)>0$.

Let $X=\{x_1,\ldots ,x_k\}$ and $X'=\{y\}$. Let us check that, for every (*A*, *B*) such that $A\subseteq X$, $B\subseteq X'$, and $|B|<|A|$,

$$\begin{aligned} \textrm{rPSD}(X)+\textrm{rPSD}(X')\geqslant \textrm{rPSD}(\tau _{A,B}(X))+\textrm{rPSD}(\tau _{B,A}(X')) \end{aligned}$$

and that the equality holds only when $(A,B)=(X,X')$. This will imply that the only $\textrm{rPSD}$-improving pair for $X,X'$ in $\mathscr {S}_{k,2}$ is $(X,X')$ itself.

Let:

- $E_0$ be the arcs in ${\uparrow }\{v_1,\ldots ,v_k\}$; that is, $(r,a_1)$ and those beginning in $\{a_1,\ldots ,a_{k-1}\}$.
- $E_1=E(N)\setminus (E_0\cup \{e_i\}_{i=1,\ldots ,k})$; that is, the arcs ending in $\{H_1,H_2,\ldots ,H_k,y\}$.

Then,

$$\begin{aligned} \textrm{rPSD}(X)=\sum _{i=1}^k w(e_i)+\sum _{e\in E_0} w(e),\quad \textrm{rPSD}(X')= \hspace{-2ex}\sum _{e\in E_0\cup E_1}\hspace{-2ex} w(e). \end{aligned}$$

Now, on the one hand, if $B=\emptyset$ and $A\ne \emptyset$

$$\begin{aligned}&\textrm{rPSD}(\tau _{A,\emptyset }(X))= \textrm{rPSD}(X\setminus A) =\sum _{x_{i}\notin A} w(e_i)+\hspace{-2ex}\sum _{e\in E_0\cap {\uparrow }(X\setminus A)}\hspace{-5ex} w(e)\\&\textrm{rPSD}(\tau _{\emptyset ,A}(X'))= \textrm{rPSD}(X'\cup A)=\textrm{rPSD}(X')+\sum _{x_{i}\in A} w(e_i) \end{aligned}$$

and then

$$\begin{aligned}&\textrm{rPSD}(X)+\textrm{rPSD}(X')-(\textrm{rPSD}(\tau _{A,\emptyset }(X))+\textrm{rPSD}(\tau _{\emptyset ,A}(X')))\\&\qquad =\sum _{e\in E_0} w(e)-\hspace{-2ex}\sum _{e\in E_0\cap {\uparrow }(X\setminus A)}\hspace{-2ex} w(e)>0 \end{aligned}$$

because for every $x_i\in A$ the arc $(a_i,v_i)$ (or $(a_{k-1},v_k)$ if $i=k$) does not belong to ${\uparrow }(X\setminus A)$ and therefore $E_0\cap {\uparrow }(X\setminus A)\subsetneq E_0$.

On the other hand, if $B=X'=\{y\}$,

$$\begin{aligned}&\textrm{rPSD}(\tau _{A,\{y\}}(X)) =\textrm{rPSD}((X\setminus A)\cup \{y\}) =\sum _{x_{i}\notin A} w(e_i)+\textrm{rPSD}(X'),\\&\textrm{rPSD}(\tau _{\{y\},A}(X')) =\textrm{rPSD}(A) =\sum _{x_{i}\in A} w(e_i)+\hspace{-1ex}\sum _{e\in E_0\cap {\uparrow }A}\hspace{-2ex} w(e) \end{aligned}$$

and then

$$\begin{aligned}&\textrm{rPSD}(X)+\textrm{rPSD}(X')-(\textrm{rPSD}(\tau _{A,\{y\}}(X))+\textrm{rPSD}(\tau _{\{y\},A}(X')))\\&\qquad =\sum _{e\in E_0} w(e)-\sum _{e\in E_0\cap {\uparrow }A} w(e)\geqslant 0, \end{aligned}$$

where, arguing as above, the inequality is an equality exactly when $A=X$.

We close this section with a refinement of Theorem 1 for level-1 networks. The proof is similar, and we provide it in Section 2 of the Supplementary file.

### Corollary 1

If *N* is a semi-*d*-ary level-1 phylogenetic network on $\Sigma$, $\textrm{rPSD}_N$ satisfies the exchange property with respect to

$$\begin{aligned} \mathscr {S}_{d}= \mathscr {S}_{0} \cup \big \{(A,\{b\})\in \mathcal {P}(\Sigma )^2:\, b\notin A,\ 1< |A|\leqslant d\big \} \end{aligned}$$

Moreover, if $X,X'$ have an improving pair $(A,\{b\})\in \mathscr {S}_{d}$, then there exists a blob in *N* with exit reticulation *H* and split node *v* such that $X\cap C(H)=\emptyset$, $b\in C(H)$, and $A\subseteq C(v)$.

## Applications

In this section we apply Theorem 1 to the study of $\textrm{rPSD}_N$-optimal subsets for low values of the level of *N* and the in-degree of its reticulations. Throughout this section, let *N* be a phylogenetic network on a set $\Sigma$ of cardinality *n* and $\textrm{rPSD}=\textrm{rPSD}_N$. We shall use the following notation:

- For every *m*, let $\textrm{Opt}_m$ be the family of $\textrm{rPSD}$-optimal subsets of $\Sigma$ of cardinality *m*:
  $$\begin{aligned} \textrm{Opt}_m = \big \{ Z\in \mathcal {P}_{m}(\Sigma ):\, \textrm{rPSD}(Z) = \max \big (\textrm{rPSD}(\mathcal {P}_{m}(\Sigma ))\big )\big \}. \end{aligned}$$
- An *optimal sequence* of *N* is a sequence $Y = (Y_m)_{0 \leqslant m \leqslant n}$ with each $Y_m \in \textrm{Opt}_m$.
- For every $k\geqslant 1$ and $d\geqslant 2$, for every $1\leqslant j\leqslant (d-1)k$, and for every $X\in \mathcal {P}(\Sigma )$,
  - $\tau _{k,d,j}(X)$ is the family of subsets of $\Sigma$ of cardinality $|X|+j$ of the form $\tau _{B,A}(X)$ (this is, $(X\setminus B)\cup A)$ with $(A,B)\in \mathscr {S}_{k,d}$, $B\subseteq X$, $A\subseteq \Sigma \setminus X$, and $|A|-|B|=j$.
  - $\textrm{Opt}\text {-}\tau _{k,d,j} (X)$ are the members of $\tau _{k,d,j}(X)$ with largest $\textrm{rPSD}$ value.
  and, analogously,
  - $\tau ^{-1}_{k,d,j}(X)$ is the family of subsets of $\Sigma$ of cardinality $|X|-j$ of the form $\tau _{A,B}(X)$ (this is, $(X\setminus A)\cup B)$ with $(A,B)\in \mathscr {S}_{k,d}$, $A\subseteq X$, $B\subseteq \Sigma \setminus X$, and $|A|-|B|=j$.
  - $\textrm{Opt}\text {-}\tau ^{-1}_{k,d,j} (X)$ are the members of $\tau ^{-1}_{k,d,j}(X)$ with largest $\textrm{rPSD}$ value.
  Notice that $X'\in \tau _{k,d,j}(X)$ if, and only if, $X\in \tau _{k,d,j}^{-1}(X')$.
- Finally, for every $k\geqslant 1$ and $d\geqslant 2$, for every $1 \leqslant j\leqslant (d-1)k$, we describe the family of subsets of $\Sigma$ of cardinality $m+j$ (resp. $m-j$) of the form $\tau _{B,A}(Y)$ (resp. $\tau _{A,B}(Y)$) with $(A,B)\in \mathscr {S}_{k,d}$, $|A|-|B|=j$, with largest $\textrm{rPSD}$ value obtained from each $Y \in \textrm{Opt}_m$:
  - $$\textrm{Opt}\text {-}\tau _{k,d,j} (\textrm{Opt}_m)= \bigcup _{Y \in \textrm{Opt}_m} \textrm{Opt}\text {-}\tau _{k,d,j}(Y)$$.
  - $$\textrm{Opt}\text {-}\tau ^{-1}_{k,d,j}(\textrm{Opt}_m)= \bigcup _{Y \in \textrm{Opt}_m} \textrm{Opt}\text {-}\tau ^{-1}_{k,d,j}(Y)$$.
  The aim of this section will be to relate each $\textrm{Opt}\text {-}\tau _{k,d,j}(\textrm{Opt}_m)$ with $\textrm{Opt}_{m+j}$ and $\textrm{Opt}\text {-}\tau _{k,d,j}^{-1}(\textrm{Opt}_{m})$ with $\textrm{Opt}_{m-j}$, providing the key ingredient of the greedy algorithm.

We begin with galled trees. As we have already mentioned, it was proved in Bordewich et al. (2022, Cor 4.6) that the optimization problem for $\textrm{rPSD}$ can be solved in polynomial time on galled trees. The next proposition strengthens this result by providing a recursive construction of the $\textrm{rPSD}$-optimal sets for these networks.

### Proposition 1

Let *N* be a galled tree. Then, for every $m=1,\ldots ,n$,

$$\begin{aligned} \textrm{Opt}_m = \textrm{Opt}\text {-}\tau _{1,2,1}(\textrm{Opt}_{m-1}). \end{aligned}$$

### Proof

Let $Y_m\in \textrm{Opt}_m$ and $Y_{m-1}\in \textrm{Opt}_{m-1}$. By Theorem 1, there exists some $(A,B)\in \mathscr {S}_{1,2}$, with $A\subseteq Y_m{\setminus } Y_{m-1}$ and $B\subseteq Y_{m-1}{\setminus } Y_m$, such that

1 $$\begin{aligned} \textrm{rPSD}(Y_m)+ \textrm{rPSD}(Y_{m-1})\leqslant \textrm{rPSD}(\tau _{A,B}(Y_m))+ \textrm{rPSD}(\tau _{B,A}(Y_{m-1})). \end{aligned}$$

Since $|A|-|B|=1$, we have that $\tau _{A,B}(Y_m)\in \mathcal {P}_{m-1}(\Sigma )$ and $\tau _{B,A}(Y_{m-1})\in \mathcal {P}_m(\Sigma )$, and then, being $Y_{m-1}$ and $Y_{m}$ optimal in $\mathcal {P}_{m-1}(\Sigma )$ and $\mathcal {P}_m(\Sigma )$, respectively,

2 $$\begin{aligned} \textrm{rPSD}(\tau _{A,B}(Y_m))\leqslant \textrm{rPSD}(Y_{m-1}),\ \textrm{rPSD}(\tau _{B,A}(Y_{m-1}))\leqslant \textrm{rPSD}(Y_m). \end{aligned}$$

Combining these inequalities with (1) we obtain

$$\begin{aligned} \begin{array}{rl} \textrm{rPSD}(Y_m)+ \textrm{rPSD}(Y_{m-1}) & \leqslant \textrm{rPSD}(\tau _{A,B}(Y_m))+\textrm{rPSD}(\tau _{B,A}(Y_{m-1})) \\ & \leqslant \textrm{rPSD}(Y_{m-1})+ \textrm{rPSD}(Y_m). \end{array} \end{aligned}$$

Then, the inequalities (2) must be equalities, from which we deduce that:

- $$\tau _{A,B}(Y_m)\in \textrm{Opt}_{m-1}$$, and thus $$Y_m=\tau _{B,A}\big (\tau _{A,B}(Y_m)\big )\in \textrm{Opt}\text {-}\tau _{1,2,1}(\textrm{Opt}_{m-1})$$.
- $$\tau _{B,A}(Y_{m-1})\in \textrm{Opt}_{m}$$, and thus $$\textrm{Opt}\text {-}\tau _{1,2,1}(Y_{m-1})\subseteq \textrm{Opt}_{m}$$.

Since the choice of the optimal sets $Y_m,Y_{m-1}$ was arbitrary, we conclude that

$$\begin{aligned} \textrm{Opt}_m \subseteq \textrm{Opt}\text {-}\tau _{1,2,1}(\textrm{Opt}_{m-1})\text { and } \textrm{Opt}\text {-}\tau _{1,2,1}(\textrm{Opt}_{m-1}) \subseteq \textrm{Opt}_m \end{aligned}$$

as stated. $$\square $$

### Remark 1

Notice that along the proof of the previous proposition we have proved that, in a galled tree, for every $Y_m\in \textrm{Opt}_{m}$ and $Y_{m-1}\in \textrm{Opt}_{m-1}$, there exists some pair $(A,B)\in \mathscr {S}_{1,2}$, with $A\subseteq Y_m{\setminus } Y_{m-1}$ and $B\subseteq Y_{m-1}{\setminus } Y_{m}$, such that $\tau _{A,B}(Y_m)\in \textrm{Opt}_{m-1}$ and $\tau _{B,A}(Y_{m-1})\in \textrm{Opt}_{m}$.

Proposition 1 implies that, on a galled tree, the members of $\textrm{Opt}_m$ are those obtained from members of $\textrm{Opt}_{m-1}$ by either optimally adding a leaf or optimally replacing a leaf by two leaves. This result yields the simple greedy polynomial time Algorithm 2 computing the family of optimal sets $\textrm{Opt}_m$ in increasing order of *m* that extends the greedy Algorithm 1 for phylogenetic trees.

**Algorithm 2:**
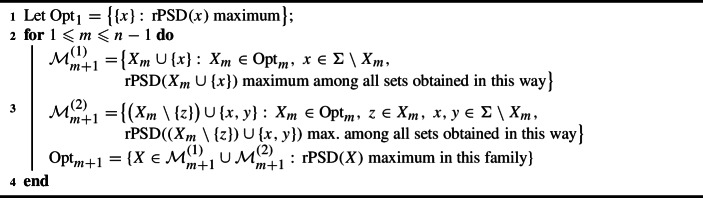
Greedy for galled trees

### Remark 2

Proposition 1 also implies that, on a galled tree, the members of each $\textrm{Opt}_{m}$ are obtained from members of $\textrm{Opt}_{m+1}$ by removing a leaf or replacing a pair of leaves by a leaf in such a way that the value of $\textrm{rPSD}$ decreases the least.

To move up in the complexity ladder of phylogenetic networks, it is convenient to introduce a notation that allows a more compact description of the arguments of the type used in the previous proposition. Given a semi-*d*-ary level-*k* phylogenetic network *N* and an optimal sequence $Y = (Y_p)_{0 \leqslant p \leqslant n}$ of it, we shall write, for every $0\leqslant q<p\leqslant n$ and for every $j\geqslant 1$,

$$\begin{aligned} (p, q) \prec \mathrel {\hspace{-2.77771pt}}\mathrel {\cdot }^Y (p-j, q+j) \end{aligned}$$

to mean that there exists an $\textrm{rPSD}$-improving pair $(A,B) \in \mathscr {S}_{k,d}$ for $Y_p$ and $Y_q$ such that $|A | - |B | = j$. When we need to emphasize an improving pair (*A*, *B*), we shall write “$(p, q) \prec \mathrel {\hspace{-2.77771pt}}\mathrel {\cdot }^Y (p-j, q+j)$ by an improving pair (*A*, *B*)”. In addition, we shall write $(p, q) \prec \mathrel {\hspace{-2.77771pt}}\mathrel {\cdot }^Y_j \{p',q'\}$ to mean that $(p,q)\prec \mathrel {\hspace{-2.77771pt}}\mathrel {\cdot }^Y (p-j,q+j)$ and $\{p-j,q+j\}=\{p',q'\}$.

### Remark 3

By Theorem 1, given any optimal sequence *Y* of a semi-*d*-ary level-*k* phylogenetic network and $0\leqslant q<p$, there always exists some $1 \leqslant j \leqslant (d-1)k$ such that $(p,q) \prec \mathrel {\hspace{-2.77771pt}}\mathrel {\cdot }^Y (p-j,\,q+j)$.

The proof of the next lemma, which we leave to the reader, is similar to that of Proposition 1; actually, that proposition is a direct consequence of this lemma for $j=1$.

### Lemma 1

Let *N* be a phylogenetic network and *Y* an optimal sequence of *N*. If $(p, q) \prec \mathrel {\hspace{-2.77771pt}}\mathrel {\cdot }^Y (p-j, q+j)$ and $\textrm{rPSD}(Y_{p-j}) + \textrm{rPSD}(Y_{q+j}) \leqslant \textrm{rPSD}(Y_{p}) + \textrm{rPSD}(Y_{q})$, then $Y_{p} \in \textrm{Opt}\text {-}\tau _{k,d,j}(\textrm{Opt}_{p-j})$ and $Y_{q} \in \textrm{Opt}\text {-}\tau _{k,d,j}^{-1}(\textrm{Opt}_{q+j})$.

In particular, if $p-q=j$ and $(p, q) \prec \mathrel {\hspace{-2.77771pt}}\mathrel {\cdot }^Y (q,p)$, then $Y_{p} \in \textrm{Opt}\text {-}\tau _{k,d,j}(\textrm{Opt}_{q})$ and $Y_{q} \in \textrm{Opt}\text {-}\tau _{k,d,j}^{-1}(\textrm{Opt}_{p})$.

### Corollary 2

Let *N* be a phylogenetic network and *Y* an optimal sequence of *N*. If there exists a closed $\prec \mathrel {\hspace{-2.77771pt}}\mathrel {\cdot }^Y$-chain of length $m \geqslant 1$

$$\begin{aligned}&(p_1,\,q_1) \prec \mathrel {\hspace{-2.77771pt}}\mathrel {\cdot }^Y_{j_1} \{p_2,\,q_2\} \text { by an improving pair} (A_1,B_1) \\&(p_2,\,q_2) \prec \mathrel {\hspace{-2.77771pt}}\mathrel {\cdot }^Y_{j_2} \{p_3,\,q_3\} \text { by an improving pair} (A_2,B_2) \\&\qquad \vdots \\&(p_m,\,q_m) \prec \mathrel {\hspace{-2.77771pt}}\mathrel {\cdot }^Y_{j_m} \{p_1,\,q_1\} \text { by an improving pair} (A_m,B_m) \end{aligned}$$

then, for each $i=1,\ldots m$,

$$\begin{aligned} Y_{p_i} \in \textrm{Opt}\text {-}\tau _{k,d,j_i}(\textrm{Opt}_{p_i-j_i})\text { and } Y_{q_i} \in \textrm{Opt}\text {-}\tau _{k,d,j_i}^{-1}(\textrm{Opt}_{q_{i}+j_i}). \end{aligned}$$

### Proof

The closed chain ensures that all the inequalities in

$$\begin{aligned}&\textrm{rPSD}(Y_{p_1}) + \textrm{rPSD}(Y_{q_1})\leqslant \textrm{rPSD}(\tau _{A_1,B_1}(Y_{p_1})) + \textrm{rPSD}(\tau _{B_1,A_1}(Y_{q_1}))\\&\quad \leqslant \textrm{rPSD}(Y_{p_2}) + \textrm{rPSD}(Y_{q_2}) \leqslant \textrm{rPSD}(\tau _{A_2,B_2}(Y_{p_2})) + \textrm{rPSD}(\tau _{B_2,A_2}(Y_{q_2}))\\&\quad \leqslant \textrm{rPSD}(Y_{p_3}) + \textrm{rPSD}(Y_{q_3}) \leqslant \textrm{rPSD}(\tau _{A_3,B_3}(Y_{p_3})) + \textrm{rPSD}(\tau _{B_3,A_3}(Y_{q_3}))\\&\quad \vdots \\&\quad \leqslant \textrm{rPSD}(Y_{p_m}) + \textrm{rPSD}(Y_{q_m}) \leqslant \textrm{rPSD}(\tau _{A_m,B_m}(Y_{p_m})) + \textrm{rPSD}(\tau _{B_m,A_m}(Y_{q_m}))\\&\quad \leqslant \textrm{rPSD}(Y_{p_1}) + \textrm{rPSD}(Y_{q_1}), \end{aligned}$$

are equalities, and the result follows from applying the Lemma 1 to each $(p_i,\,q_i) \prec \mathrel {\hspace{-2.77771pt}}\mathrel {\cdot }^Y (p_i-j_i,\, q_i+j_i).$ $\square$

It is time to move one step up in the complexity ladder of phylogenetic networks. Recall that

$$\begin{aligned} \mathscr {S}_{2,2} =\mathscr {S}_{1,3}=\mathscr {S}_{0}\cup \{(A,B)\in {\mathcal {P}}(\Sigma )^2:A\cap B=\emptyset ,\ 1\leqslant |B|<|A|\leqslant 3)\} \end{aligned}$$

and in particular, for every $j=1,2$, $\textrm{Opt}\text {-}\tau _{1,3,j}=\textrm{Opt}\text {-}\tau _{2,2,j}$.

### Proposition 2

If *N* is a semibinary level-2 or a semi-3-ary level-1 network, then:

- $$\textrm{Opt}_m \subseteq \textrm{Opt}\text {-}\tau _{2,2,1}(\textrm{Opt}_{m-1}) \cup \textrm{Opt}\text {-}\tau _{2,2,2}(\textrm{Opt}_{m-2})$$ for every $$m=1,\ldots ,n$$.
- $$\textrm{Opt}_{m} \subseteq \textrm{Opt}\text {-}\tau _{2,2,1}^{-1}(\textrm{Opt}_{m+1}) \cup \textrm{Opt}\text {-}\tau _{2,2,2}^{-1}(\textrm{Opt}_{m+2})$$ for every $$m=1,\ldots ,n-1$$.

### Proof

Let *Y* be an optimal sequence of *N* and fix $1 \leqslant m \leqslant n$. Then, by Theorem 1,

3 $$\begin{aligned} (m, m-1) \prec \mathrel {\hspace{-2.77771pt}}\mathrel {\cdot }^Y (m-j_1, m-1+j_1) \end{aligned}$$

for some $j_1 = 1$ or $j_1 = 2$.

1. If $j_1 = 1$, Eqn. (3) says that $(m, m-1) \prec \mathrel {\hspace{-2.77771pt}}\mathrel {\cdot }^Y (m-1, m)$, and hence, by Corollary 2,
  $$\begin{aligned} Y_m \in \textrm{Opt}\text {-}\tau _{2,2,1}(\textrm{Opt}_{m-1})\text { and } Y_{m-1} \in \textrm{Opt}\text {-}\tau _{2,2,1}^{-1}(\textrm{Opt}_m). \end{aligned}$$
2. If $j_1 = 2$, Eqn. (3) says that $(m, m-1) \prec \mathrel {\hspace{-2.77771pt}}\mathrel {\cdot }^Y (m-2, m+1)$. Applying Theorem 1 again,
  $$\begin{aligned} (m+1, m-2) \prec \mathrel {\hspace{-2.77771pt}}\mathrel {\cdot }^Y (m+1-j_2, m-2+j_2), \end{aligned}$$
  for some $j_2 = 1$ or $j_2 = 2$. In both cases, $\{m+1-j_2, m-2+j_2\}=\{m-1,m\}$, thus closing the $\prec \mathrel {\hspace{-2.77771pt}}\mathrel {\cdot }$-chain initiated with (3). Then, by Corollary 2,
  $$\begin{aligned} Y_m \in \textrm{Opt}\text {-}\tau _{2,2,2}(\textrm{Opt}_{m-2})\text { and }Y_{m-1} \in \textrm{Opt}\text {-}\tau _{2,2,2}^{-1}(\textrm{Opt}_{m+1}). \end{aligned}$$

Thus, in both cases we have that

$$\begin{aligned} \begin{array}{l} Y_m \in \textrm{Opt}\text {-}\tau _{2,2,1}(\textrm{Opt}_{m-1}) \cup \textrm{Opt}\text {-}\tau _{2,2,2}(\textrm{Opt}_{m-2}),\\ Y_{m-1} \in \textrm{Opt}\text {-}\tau _{2,2,1}^{-1}(\textrm{Opt}_m) \cup \textrm{Opt}\text {-}\tau _{2,2,2}^{-1}(\textrm{Opt}_{m+1}), \end{array} \end{aligned}$$

which, by the arbitrary choice of *Y* and *m*, concludes the proof. $\square$

Point (a) in the last proposition tells us that if *N* is semibinary level-2 or semi-3-ary level-1, all members of each $\textrm{Opt}_m$ are obtained either from members of $\textrm{Opt}_{m-1}$ by optimally adding a leaf, optimally replacing a leaf by a pair of leaves, or optimally replacing a pair of leaves by a triple of leaves (this possibility need not be considered in the semi-3-ary level-1 case by Corollary 1), or from members of $\textrm{Opt}_{m-2}$ by optimally replacing a leaf by a triple of leaves. This proves the correctness of the polynomial time greedy Algorithm 3 to compute the family of optimal sets $\textrm{Opt}_m$ for such a network *N* in increasing order of *m* (as we have mentioned, if *N* is semi-3-ary level-1, the sets ${\mathcal {M}}^{(4)}$ in the loop need not be computed).

**Algorithm 3:**
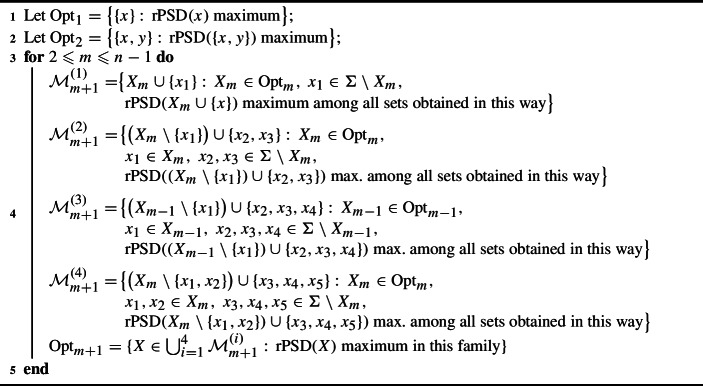
Greedy for semibinary level-2 or semi-3-ary level-1 networks

**Fig. 3:**
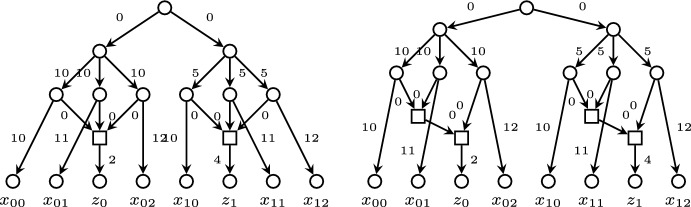
The networks in Example 3

### Example 3

Consider the phylogenetic networks in Fig. 3. On the left, a semi-3-ary level-1 network and on the right a semibinary level-2 network obtained by blowing up the reticulations in the left-hand side network into a pair of in-degree 2 connected reticulations. In both networks, we have the following optimal sets of leaves:

$$\begin{aligned} \begin{array}{ll} \textrm{Opt}_1\!:\ \{z_0\} & \qquad \textrm{Opt}_5\!:\ \{x_{00},x_{01},x_{02},x_{11},x_{12}\} \\ \textrm{Opt}_2\!:\ \{z_0, z_1\}& \qquad \textrm{Opt}_6\!:\ \{x_{00},x_{01},x_{02},x_{10},x_{11},x_{12}\} \\ \textrm{Opt}_3\!: \ \{x_{11},x_{12},z_0\} & \qquad \textrm{Opt}_7\!: \ \{x_{00},x_{01},x_{02},x_{10},x_{11},x_{12},z_1\}\\ \textrm{Opt}_4\!:\ \{x_{00},x_{01},x_{02},z_1\} \end{array} \end{aligned}$$

Then, in both networks,

$$\begin{aligned} \{x_{00},x_{01},x_{02},z_1\}\! \in \! \textrm{Opt}_4\! \setminus \! \textrm{Opt}\text {-}\tau _{2,2,1}(\textrm{Opt}_3),\ \{x_{11},x_{12},z_0\}\! \in \! \textrm{Opt}_3\! \setminus \! \textrm{Opt}\text {-}\tau _{2,2,1}^{-1}(\textrm{Opt}_4). \end{aligned}$$

Now, if we move one more step further in the complexity ladder, the structure of the optimal sets is no longer so simple.

### Proposition 3

If *N* is a semibinary level-3 or a semi-4-ary level-1 network, then, for every $m=1,\ldots ,n$, at least one of the following assertions is true:

- $$\textrm{Opt}_m \subseteq \bigcup _{j=1}^3 \textrm{Opt}\text {-}\tau _{k,d,j}(\textrm{Opt}_{m-j})$$ and $$\textrm{Opt}_{m-1} \subseteq \bigcup _{j=1}^3 \textrm{Opt}\text {-}\tau _{k,d,j}^{-1}(\textrm{Opt}_{m-1+j})$$.
- $$\textrm{Opt}_{m+1} = \textrm{Opt}\text {-}\tau _{k,d,3}(\textrm{Opt}_{m-2})$$,

where (*k*, *d*) is (3, 2) or (1, 4), depending on the type of network.

### Proof

To begin with, notice that

$$\begin{aligned} \mathscr {S}_{3,2}&=\mathscr {S}_{0}\cup \{(A,B)\in {\mathcal {P}}(\Sigma )^2 : A\cap B=\emptyset , 1\leqslant |B|<|A|< 6,\ |A|-|B|\leqslant 3\}\\ \mathscr {S}_{1,4}&=\mathscr {S}_{0}\cup \{(A,B)\in {\mathcal {P}}(\Sigma )^2 : A\cap B=\emptyset , 1\leqslant |B|<|A|\leqslant 4\} \end{aligned}$$

and therefore $\mathscr {S}_{1,4}\subseteq \mathscr {S}_{3,2}$. To simplify the notation, we shall abbreviate $\textrm{Opt}\text {-}\tau _{k,d,j}$ by simply $\textrm{Opt}\text {-}\tau _{j}$. Observe that *j* can only go from 1 to 3.

**Fig. 4:**
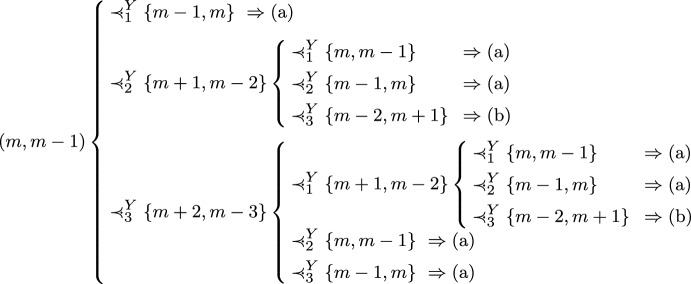
Sketch of the proof of Proposition 3

Let *Y* be an optimal sequence of *N* and fix $1 < m \leqslant n$. To ease the task of the reader, we sketch the flow of the proof in Fig. 4; all implications leading to (a) or (b) are due to Cor. 2.

By Theorem 1,

4 $$\begin{aligned} (m, m-1) \prec \mathrel {\hspace{-2.77771pt}}\mathrel {\cdot }^Y (m-j_1,\,m-1+j_1) \end{aligned}$$

for some $$j_1 \in \{ 1,2,3\}$$.

1. If $j_1=1$, then $(m, m-1) \prec \mathrel {\hspace{-2.77771pt}}\mathrel {\cdot }^Y (m-1,\, m)$ and we conclude as in (1) in the proof of Proposition 2 that $Y_m \in \textrm{Opt}\text {-}\tau _{1}(\textrm{Opt}_{m-1})$ and $Y_{m-1} \in \textrm{Opt}\text {-}\tau _{1}^{-1}(\textrm{Opt}_m)$.
2. If $j_1 = 2$, then $(m, m-1) \prec \mathrel {\hspace{-2.77771pt}}\mathrel {\cdot }^Y (m-2, m+1)$. Applying Theorem 1 again,
  $$\begin{aligned} (m+1, m-2) \prec \mathrel {\hspace{-2.77771pt}}\mathrel {\cdot }^Y (m+1-j_2, m-2+j_2), \end{aligned}$$
  for some $$j_2 \in \{ 1,2,3\}$$.
  - If $j_2 = 1$ or $j_2 = 2$, $(m+1, m-2) \prec \mathrel {\hspace{-2.77771pt}}\mathrel {\cdot }_{j_2}^Y \{m,m-1\}$ and we conclude as in (2) in the proof of Proposition 2 that $Y_m \in \textrm{Opt}\text {-}\tau _{2}(\textrm{Opt}_{m-2})$ and $Y_{m-1} \in \textrm{Opt}\text {-}\tau _{2}^{-1}(\textrm{Opt}_{m+1})$.
  - When $j_2 = 3$, we have $(m+1, m-2) \prec \mathrel {\hspace{-2.77771pt}}\mathrel {\cdot }^Y (m-2, m+1)$ and we can only deduce that $Y_{m+1} \in \textrm{Opt}\text {-}\tau _{3}(\textrm{Opt}_{m-2})$ and $Y_{m-2} \in \textrm{Opt}\text {-}\tau _{3}^{-1}(\textrm{Opt}_{m+1})$.
3. If $j_1 = 3$, then $(m, m-1) \prec \mathrel {\hspace{-2.77771pt}}\mathrel {\cdot }^Y (m-3, m+2)$. Applying Theorem 1 again,
  $$\begin{aligned} (m+2, m-3) \prec \mathrel {\hspace{-2.77771pt}}\mathrel {\cdot }^Y (m+2-j_2, m-3+j_2), \end{aligned}$$
  for some $$j_2 \in \{ 1,2,3\}$$.
  - If $j_2 = 1$, then $(m+2, m-3) \prec \mathrel {\hspace{-2.77771pt}}\mathrel {\cdot }^Y (m+1, m-2)$. Applying Theorem 1, we have
    $$\begin{aligned} (m+1, m-2) \prec \mathrel {\hspace{-2.77771pt}}\mathrel {\cdot }^Y (m+1-j_3, m-2+j_3) \end{aligned}$$
    for some $$j_3 \in \{ 1,2,3\}$$.
    - If $j_3 = 1$ or $j_3=2$, then $(m+1, m-2) \prec \mathrel {\hspace{-2.77771pt}}\mathrel {\cdot }^Y \{m, m-1\}$, closing the $\prec \mathrel {\hspace{-2.77771pt}}\mathrel {\cdot }$-chain initiated with (4). Then, by Corollary 2, $Y_m \in \textrm{Opt}\text {-}\tau _{3}(\textrm{Opt}_{m-3})$ and $Y_{m-1} \in \textrm{Opt}\text {-}\tau _{3}^{-1}(\textrm{Opt}_{m+2})$.
    - If $j_3 = 3$, then $(m+1, m-2) \prec \mathrel {\hspace{-2.77771pt}}\mathrel {\cdot }^Y (m-2, m+1)$ as in (2.b) and we only have that $Y_{m+1} \in \textrm{Opt}\text {-}\tau _{3}(\textrm{Opt}_{m-2})$ and $Y_{m-2} \in \textrm{Opt}\text {-}\tau _{3}^{-1}(\textrm{Opt}_{m+1})$.
  - If $j_2 = 2$ or $j_2 = 3$, then $(m+2, m-3) \prec \mathrel {\hspace{-2.77771pt}}\mathrel {\cdot }^Y \{m, m-1\}$, closing the $\prec \mathrel {\hspace{-2.77771pt}}\mathrel {\cdot }$-chain initiated with (4). Then, by Corollary 2, $Y_m \in \textrm{Opt}\text {-}\tau _{3}(\textrm{Opt}_{m-3})$ and $Y_{m-1} \in \textrm{Opt}\text {-}\tau _{3}^{-1}(\textrm{Opt}_{m+2})$.

Summarizing, we only have two possibilities:

- On the one hand, in the cases (1), (2.a), (3.a.i), and (3.b),
  $$\begin{aligned} Y_m \in \bigcup _{j=1}^3 \textrm{Opt}\text {-}\tau _{j}(\textrm{Opt}_{m-j})\text { and } Y_{m-1} \in \bigcup _{j=1}^3 \textrm{Opt}\text {-}\tau _{j}^{-1}(\textrm{Opt}_{m-1+j}). \end{aligned}$$
- On the other hand, in the cases (2.b) and (3.a.ii),
  $$\begin{aligned} Y_{m+1} \in \textrm{Opt}\text {-}\tau _{3}(\textrm{Opt}_{m-2})\text { and } \textrm{Opt}\text {-}\tau _{3}(Y_{m-2}) \subseteq \textrm{Opt}_{m+1}. \end{aligned}$$

By the arbitrary choice of *Y* and *m*, this concludes the proof. $\square$

A similar result holds for (*k*, *d*) such that $(d-1)k=4$. We give its proof in Section 3 of the Supplementary file.

### Proposition 4

If *N* is a semi-5-ary level-1 or a semi-3-ary level-2 network, then, for every $m=1,\ldots ,n$, at least one of the following assertions is true:

- $$\textrm{Opt}_m \subseteq \bigcup _{j=1}^4 \textrm{Opt}\text {-}\tau _{k,d,j}(\textrm{Opt}_{m-j})$$ and $$\textrm{Opt}_{m-1} \subseteq \bigcup _{j=1}^4 \textrm{Opt}\text {-}\tau _{k,d,j}^{-1}(\textrm{Opt}_{m-1+j})$$.
- $$\textrm{Opt}_{m+1} = \textrm{Opt}\text {-}\tau _{k,d,3}(\textrm{Opt}_{m-2})$$,

where $(k,d)=(2,3)$ or (1, 5), depending on the type of network.

So, while we could give a greedy optimization algorithm for semibinary level-2 networks or semi-3-ary level-1 networks, an analogous argument fails for more complex networks. The reason why Propositions 3 and 4 are not sufficient to provide such a greedy algorithm is that we would require their assertion (a) —or a similar expression— to be true for all *m*. In the occurrence of any *m* where only assertion (b) holds, we do not have enough information about $\textrm{Opt}_m$ to be able to ensure that it can be obtained from previous optimal sets.

### Remark 4

A close analysis of the proof of Proposition 3, using Corollary 2 in its full strength, shows that we actually have a more general result: for every optimal sequence *Y* of *N* and for every $1 < m \leqslant n$, at least one of the following conditions holds (the labels correspond to the cases in the proof):

- $$Y_m \in \textrm{Opt}\text {-}\tau _{1}(\textrm{Opt}_{m-1})$$ and $$Y_{m-1} \in \textrm{Opt}\text {-}\tau _{1}^{-1}(\textrm{Opt}_m)$$.
- $$Y_m \in \textrm{Opt}\text {-}\tau _{2}(\textrm{Opt}_{m-2})$$, $$Y_{m-1}\in \textrm{Opt}\text {-}\tau _{2}^{-1} (\textrm{Opt}_{m+1})$$, and
  $$Y_{m+1}\in \textrm{Opt}\text {-}\tau _{1}(\textrm{Opt}_{m})$$ and $$Y_{m-2}\in \textrm{Opt}\text {-}\tau _{1}^{-1}\textrm{Opt}_{m-1}$$, or
  $$Y_{m+1}\in \textrm{Opt}\text {-}\tau _{2}(\textrm{Opt}_{m-1})$$ and $$Y_{m-2}\in \textrm{Opt}\text {-}\tau _{2}^{-1}\textrm{Opt}_{m}$$.
- $$Y_{m+1} \in \textrm{Opt}\text {-}\tau _{3}(\textrm{Opt}_{m-2})$$ and $$Y_{m-2} \in \textrm{Opt}\text {-}\tau _{3}^{-1}(\textrm{Opt}_{m+1})$$.
- $$Y_m \in \textrm{Opt}\text {-}\tau _{3}(\textrm{Opt}_{m-3})$$, $$Y_{m-1}\in \textrm{Opt}\text {-}\tau _{3}^{-1} (\textrm{Opt}_{m+2})$$, $$Y_{m+2}\in \textrm{Opt}\text {-}\tau _{1}(\textrm{Opt}_{m+1})$$, $$Y_{m-3}\in \textrm{Opt}\text {-}\tau _{1}^{-1}\textrm{Opt}_{m-2}$$, and
  $$Y_{m+1}\in \textrm{Opt}\text {-}\tau _{1}(\textrm{Opt}_{m})$$ and $$Y_{m-2}\in \textrm{Opt}\text {-}\tau _{1}^{-1}\textrm{Opt}_{m-1}$$, or
  $$Y_{m+1}\in \textrm{Opt}\text {-}\tau _{2}(\textrm{Opt}_{m-1})$$ and $$Y_{m-2}\in \textrm{Opt}\text {-}\tau _{2}^{-1}\textrm{Opt}_{m}$$.
- $$Y_{m+1} \in \textrm{Opt}\text {-}\tau _{3}(\textrm{Opt}_{m-2})$$ and $$Y_{m-2} \in \textrm{Opt}\text {-}\tau _{3}^{-1}(\textrm{Opt}_{m+1})$$.
- $$Y_m \in \textrm{Opt}\text {-}\tau _{3}(\textrm{Opt}_{m-3})$$, $$Y_{m-1} \in \textrm{Opt}\text {-}\tau _{3}^{-1}(\textrm{Opt}_{m+2})$$, and
  $$Y_{m+2}\in \textrm{Opt}\text {-}\tau _{2}(\textrm{Opt}_{m})$$ and $$Y_{m-3}\in \textrm{Opt}\text {-}\tau _{2}^{-1}\textrm{Opt}_{m-1}$$, or
  $$Y_{m+2}\in \textrm{Opt}\text {-}\tau _{3}(\textrm{Opt}_{m-1})$$ and $$Y_{m-3}\in \textrm{Opt}\text {-}\tau _{3}^{-1}\textrm{Opt}_{m}$$.

Unfortunately, the extra information obtained in this way is still not enough to prove the correctness of a greedy $\textrm{rPSD}$-optimization algorithm for the networks considered in that proposition. A similar situation appears in the context of Proposition 4.

But we must point out that we have not been able to find any semibinary level-3 or any semi-4-ary level-1 network for which $\textrm{Opt}_m \nsubseteq \bigcup _{j=1}^3 \textrm{Opt}\text {-}\tau _{k,d,j}(\textrm{Opt}_{m-j})$ for some *m*. Similarly, we have not been able to find any semi-5-ary level-1 or any semi-3-ary level-2 network for which $\textrm{Opt}_m \nsubseteq \bigcup _{j=1}^4 \textrm{Opt}\text {-}\tau _{k,d,j}(\textrm{Opt}_{m-j})$ for some *m*. So, it might be possible that the greedy algorithm also works in these cases, since we have not discovered a counterexample that disproves its correctness for these types of networks. In Section 4 of the Supplementary file we provide several examples that illustrate our search for a counterexample. More examples can be found in the second author’s PhD Thesis (Riera 2023).

## Conclusions

PD on phylogenetic trees satisfies the strong exchange property that guarantees that, for every two sets of leaves of different cardinalities, a leaf can always be moved from the larger set to the smaller one without decreasing the sum of the PD values. But rPSD does not longer satisfy this exchange property even for galled trees. In this paper we have generalized this exchange property to rPSD on phylogenetic networks of bounded level and reticulations’ in-degree, showing that a similar results holds if we allow more involved exchanges of leaves’ subsets. Our final goal was to use this generalized exchange property to find a polynomial time greedy algorithm for the optimization of rPSD on phylogenetic networks of bounded level and in-degree of reticulations. We have ultimately failed in this goal. We have indeed shown that the generalized exchange property entails such a greedy algorithm for semibinary level-2 networks and semi-3-ary level-1 networks (and sheds new light on the structure of the families of rPSD-optimal sets $\textrm{Opt}_m$ on galled trees) but it cannot be used, as it stands, to obtain such an algorithm on more complex networks. However, we have not been able to find examples of semibinary level-3 networks or semi-4-ary level-1 networks where the greedy algorithm fails: it is simply that the generalized exchange property alone seems not to be enough to prove its correctness.

Finally, it is important to point out that just like the $\textrm{rPSD}$ optimization problem itself, testing counterexamples is computationally expensive, too. While the greedy algorithm runs in polynomial time, finding whether $\textrm{Opt}_m$ can be obtained from some $\textrm{Opt}_{m-j}$ or not still requires calculating $\textrm{Opt}_m$ by brute force, and testing whether the exchange property holds for a certain subset of $\mathscr {S}_{k,d}$ where $|A | - |B | < j$ also requires testing all subsets $X,X' \subseteq \Sigma$. All these operations are exponential, hence trying even slightly larger examples can dramatically increase the runtime of the test.

## Supplementary Information

Below is the link to the electronic supplementary material.

Supplementary file 1 (pdf 487 KB)

## Figures and Tables

**Fig. 5:**
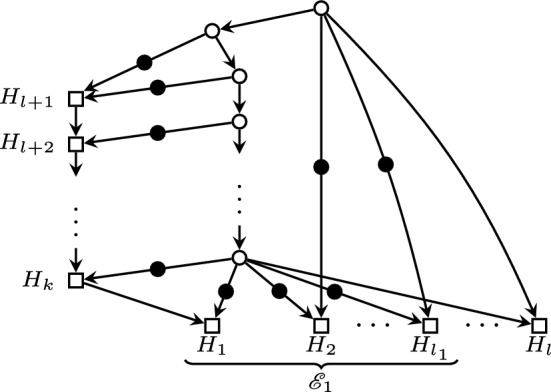
A semibinary *k*-blob with *l* exit reticulations, a subset $\mathscr {E}_1$ of $l_1>0$ exit reticulations, and an independent set of nodes (represented by filled circles) without descendant exit reticulations outside $\mathscr {E}_1$ reaching the upper bound in Lemma 2 for $d=2$

**Fig. 6:**
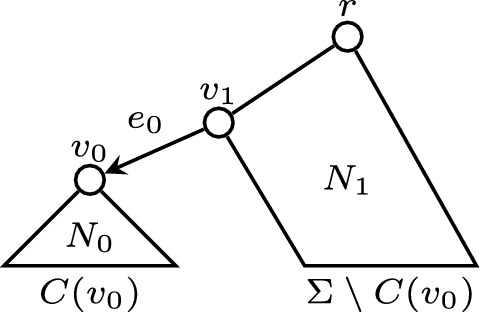
The network *N* in case (a)

**Fig. 7:**
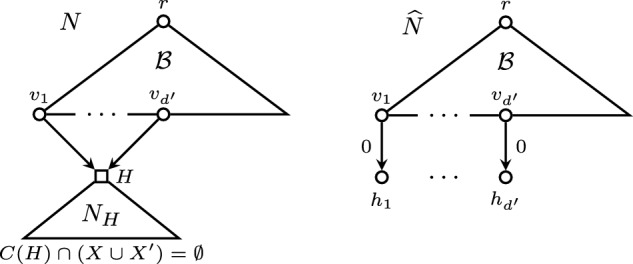
The networks *N* and $\widehat{N}$ in case (c.1)

## Appendix A: Proof of Theorem 1

We begin by stating two auxiliary lemmas. From now on, we shall call a *semi*-*d*-*ary* *k*-*blob* any blob with *k* reticulations, all of them of in-degree $\leqslant d$. Given such a semi-*d*-ary *k*-blob ${\mathcal {B}}$ and a non-empty subset $\mathscr {E}_1$ of its exit reticulations, the first lemma provides a sharp upper bound for the cardinality of any independent set of nodes *V* of ${\mathcal {B}}$ whose members have no descendant exit reticulation outside $\mathscr {E}_1$. This bound will entail the bound for the cardinality of *A* in the definition of $\mathscr {S}_{k,d}$. We give the proof of this lemma in Section 1 of the Supplementary file.

### Lemma 2

Let ${\mathcal {B}}$ be a semi-*d*-ary *k*-blob with *l* exit reticulations and $\mathscr {E}_1$ a non-empty subset of its exit reticulations of cardinality $l_1\geqslant 1$. Then, for every independent set of nodes *V* of ${\mathcal {B}}$ without descendant exit reticulations outside $\mathscr {E}_1$, $|V|\leqslant dl_1+(d-1)(k-l).$

The constructions explained in the proof of this lemma easily show that the bound it provides is sharp, in the sense that, for every $d,k,l,l_1$ with $d\geqslant 2$ and $k\geqslant l\geqslant l_1\geqslant 1$, there are semi-*d*-ary *k*-blobs with *l* exit reticulations and subsets $\mathscr {E}_1$ of $l_1$ exit reticulations containing an independent set of nodes *V* without descendant exit reticulations outside $\mathscr {E}_1$ of cardinality $dl_1+(d-1)(k-l)$: cf. Fig. 5.

### Remark 5

By Lemma 2, if ${\mathcal {B}}$ is a semi-*d*-ary blob without internal reticulations, if $\mathscr {E}_1$ is a subset of its exit reticulations of cardinality $l_1$, and if *V* is an independent set of nodes in ${\mathcal {B}}$ without descendant exit reticulations outside $\mathscr {E}_1$, then $|V|\leqslant l_1d$. A close analysis of the proof of that lemma easily shows that the upper bound $|V|=dl_1$ is achieved when all the reticulations in $\mathscr {E}_1$ have in-degree *d* and the set *V* contains, for every $H\in \mathscr {E}_1$, exactly *d* nodes whose only reticulate descendant is *H*. Of course, such sets do not always exist: for instance, when ${\mathcal {B}}$ contains a node that is a parent of two different exit reticulations.

The second auxiliary lemma extracts a key technical step in the proof of Theorem 1. This lemma provides an analog of the exchange property for sets of ancestors of multisets of nodes of a blob. More precisely, we prove that if $X,X'$ are multisets of nodes of a semi-*d*-ary *k*-blob with $|X|>|X'|$ and satisfying some extra conditions (those under which we shall apply the lemma in the proof of the main theorem) then there exist a subset *A* of *X* disjoint with $X'$ and a submultiset *B* of $X'$ disjoint with *X* whose cardinalities satisfy the restrictions defining the family $\mathscr {S}_{k,d}$ and such that if we replace *X* and $X'$ by $(X{\setminus } A)\cup B$ and $(X'{\setminus } B)\cup A$, the set of nodes that are simultaneously ancestors of nodes in both sets does not decrease.

We use in this lemma some standard notation for multisets *X*: $m_X(v)$ denotes the multiplicity of an element *v* in *X*; ${{\,\textrm{Supp}\,}}X$ denotes the *support* of *X*, that is, the set of elements *v* such that $m_{ X}(v) > 0$; we say that *X* is a *set* when all its multiplicities are $\leqslant 1$, and then we identify it with its support; a submultiset *Y* of *X* is *full* when $m_Y(y)=m_X(y)$ for every $y\in {{\,\textrm{Supp}\,}}Y\subseteq {{\,\textrm{Supp}\,}}X$; and the cardinality of *X* is $|X |=\sum _{v\in {{\,\textrm{Supp}\,}}X} m_X(v)$. We shall also use the notation $\tau _{ S, T}( X)=( X{\setminus } S)\cup T$ when *X*, *S*, *T* are multisets with $S \subseteq X$ and ${{\,\textrm{Supp}\,}}T \subseteq \Sigma {\setminus } {{\,\textrm{Supp}\,}}X$.

This lemma also uses some basic properties of ${\uparrow }$-notation. Some simple results in this regard are that, for any two sets *A*, *B*, ${\uparrow }(A\cup B)={\uparrow }A\cup {\uparrow }B$ and ${\uparrow }A {\setminus } {\uparrow }B \subseteq {\uparrow }(A {\setminus } B)$, and that if $A \subseteq B$, then ${\uparrow }A \subseteq {\uparrow }B$. Moreover, given a multiset *A*, we define ${\uparrow }A$ as ${\uparrow }{{\,\textrm{Supp}\,}}A$, without taking into account the multiplicities of the elements of *A*.

### Lemma 3

Let $${\mathcal {B}}$$ be a semi-*d*-ary *k*-blob and $$ X, {X'}$$ two multisets of nodes of $${\mathcal {B}}$$ with $$|X' | < |X |$$ and satisfying the following two further conditions:

(i) For each $v \in V({\mathcal {B}})$, if $m_{X'}(v) < m_{X}(v)$, then $m_X(v) = 1$ and $m_{X'}(v) = 0$.
(ii) Each exit reticulation of ${\mathcal {B}}$ belongs to *X* or $X'$.

Then

5 $$\begin{aligned} {\uparrow }X\cap {\uparrow }X'\subseteq {\uparrow }\tau _{A,B}(X)\cap {\uparrow }\tau _{B,A}(X') \end{aligned}$$

for some set $A \subseteq {{\,\textrm{Supp}\,}}X \setminus {{\,\textrm{Supp}\,}}X'$ and some full submultiset *B* of $X'$ with ${{\,\textrm{Supp}\,}}B \subseteq {{\,\textrm{Supp}\,}}X' {\setminus } {{\,\textrm{Supp}\,}}X$ such that $B=\emptyset$ and $|A | = 1$, or $0< |B | < |A | = d$, or $0< |B |< |A | < dk$ and $|A | - |B | \leqslant (d-1)k$.

### Proof

First, we introduce some notation.

- Let $\mathscr {E}$ be the set of exit reticulations of ${\mathcal {B}}$, and let
  $$\begin{aligned} \begin{array}{c} \mathscr {E}_X = \mathscr {E}\cap ({{\,\textrm{Supp}\,}}X \setminus {{\,\textrm{Supp}\,}}X'),\ \mathscr {E}_{X'} = \mathscr {E}\cap ({{\,\textrm{Supp}\,}}X' \setminus {{\,\textrm{Supp}\,}}X),\\[1ex] \mathscr {E}_{X,X'} = \mathscr {E}\cap ({{\,\textrm{Supp}\,}}X \cap {{\,\textrm{Supp}\,}}X'). \end{array} \end{aligned}$$
  Let $l_X = |\mathscr {E}_X |$, $l_{X'} = |\mathscr {E}_{X'} |$ and $l_{X,X'} = |\mathscr {E}_{X,X'} |$. By (ii), $l_X + l_{X'} + l_{X,X'} = |\mathscr {E} |$.
- For each $H \in \mathscr {E}$, let $\uparrow _\textit{only}\, H$ be the set ${\uparrow }H {\setminus } {\uparrow }(\mathscr {E}{\setminus } \{H\})$ of nodes whose only descendant exit reticulation is *H*. Since every node in $V({{\mathcal {B}}})$ has some descendant exit reticulation, $\uparrow _\textit{only}\, H=V({\mathcal {B}}) {\setminus } {\uparrow }(\mathscr {E}{\setminus } \{H\})$. Observe that $\uparrow _\textit{only}\, H\cap \uparrow _\textit{only}\, H'=\emptyset$ if $H\ne H'$.
- Let ${\widehat{X}}$ be the set ${{\,\textrm{Supp}\,}}X \setminus {{\,\textrm{Supp}\,}}X'$. By (i), ${\widehat{X}}= \{ v \in V({{\mathcal {B}}}):\, m_{X'}(v) < m_X(v) \}$.
- Let ${\widehat{X}}'$ be the full submultiset of $X'$ supported on ${{\,\textrm{Supp}\,}}X' \setminus {{\,\textrm{Supp}\,}}X$.

The inequality $|X|>|X'|$ implies that $|{\widehat{X}} | > |{\widehat{X}}' |$, too. Indeed:

6 $$\begin{aligned} 0&< |X | - |X' | = \sum _{v \in V({\mathcal {B}})} (m_X(v) - m_{X'}(v))\nonumber \\&= \hspace{-2ex}\sum _{\begin{array}{c} {v \in V({\mathcal {B}})} \\ m_X(v)> m_{X'}(v) \end{array}}\hspace{-2ex} (m_X(v) - m_{X'}(v)) -\hspace{-2ex} \sum _{\begin{array}{c} {v \in V({\mathcal {B}})} \\ m_{X'}(v)> m_X(v) \end{array}}\hspace{-2ex} (m_{X'}(v) - m_X(v)) \nonumber \\&= |{\widehat{X}} | - \hspace{-2ex}\sum _{\begin{array}{c} {v \in V({\mathcal {B}})} \\ m_{X'}(v) > m_X(v) \end{array}}\hspace{-2ex} (m_{X'}(v) - m_X(v)) \leqslant |{\widehat{X}} | - \sum _{v \in {\widehat{X}}'} (m_{X'}(v) - m_X(v)) \nonumber \\&= |{\widehat{X}} | - \sum _{v \in {\widehat{X}}'} m_{X'}(v) = |{\widehat{X}} | - |{\widehat{X}}' |. \end{aligned}$$

We shall consider three cases; in all of them we shall choose a subset $A\subseteq \widehat{X}$ and a full submultiset $B\subseteq \widehat{X}'$ satisfying the requirements in the statement and we shall prove that they satisfy Eqn. (5).

**(a)** If there exists some $x \in {\widehat{X}}$ with a proper descendant in *X*, then $x\in {\uparrow }(X\setminus \{x\})$ and hence ${\uparrow }X={\uparrow }(X\setminus \{x\})$. In this case, taking $A = \{x\}$ and $B = \emptyset$ we have that

$$\begin{aligned} {\uparrow }X\cap {\uparrow }X'={\uparrow }(X\setminus \{x\})\cap {\uparrow }X'\subseteq {\uparrow }(X\setminus \{x\})\cap {\uparrow }(X' \cup \{x\}). \end{aligned}$$

**(b)** Assume that no $x \in {\widehat{X}}$ has any proper descendant in *X* and that $\mathscr {E}_{X'} = \emptyset$. This implies that $\mathscr {E}= \mathscr {E}_X \cup \mathscr {E}_{X,X'} \subseteq X$ and that ${\widehat{X}} = \mathscr {E}_X$, as any $x \in {\widehat{X}} {\setminus } \mathscr {E}_X$ would have some proper descendant in $\mathscr {E}\subseteq X$.

In this case, there exists an $H_0 \in \mathscr {E}_X$ such that $\widehat{X}'\subseteq {\uparrow }(\mathscr {E}{\setminus } \{H_0\})$. Indeed, assume that for every $H \in \mathscr {E}_X$ there existed some node $x'_H\in {\widehat{X}}'$ without any descendant in $\mathscr {E}\setminus \{H\}$. Then, each $x'_H$ would belong to $\uparrow _\textit{only}\, {H}$. Since the sets $\uparrow _\textit{only}\, {H}$ are pairwise disjoint, the nodes $x'_H$ would be pairwise different, forming a subset of ${{\,\textrm{Supp}\,}}{\widehat{X}}'$ of cardinality $|\mathscr {E}_X|=|\widehat{X}|$, which cannot exist because $|\widehat{X}' |< |{\widehat{X}} |$.

Take then $A = \{H_0\}$ and $B = \emptyset$. If can prove that ${\uparrow }X' \subseteq {\uparrow }(X\setminus \{H_0\})$, then we will have

$$\begin{aligned} {\uparrow }X\cap {\uparrow }X' \subseteq {\uparrow }X' = {\uparrow }(X\setminus \{H_0\})\cap {\uparrow }X' \subseteq {\uparrow }(X\setminus \{H_0\})\cap {\uparrow }(X'\cup \{H_0\}). \end{aligned}$$

So, let $v \in {\uparrow }X'$. There are two possibilities:

- If *v* has some descendant in ${\widehat{X}}'$, then the latter will have a descendant in $\mathscr {E}{\setminus } \{H_0\}\subseteq X{\setminus } \{H_0\}$, which will also be a descendant of *v*.
- If *v* has no descendant in ${\widehat{X}}'$, then
  $$\begin{aligned} v \in {\uparrow }X' \setminus {\uparrow }{\widehat{X}}' \subseteq {\uparrow }(X' \setminus {\widehat{X}}')= &  {\uparrow }(X \cap X') = {\uparrow }(X \setminus {\widehat{X}}) \\= &  {\uparrow }(X \setminus \mathscr {E}_X) \subseteq {\uparrow }(X \setminus \{H_0\}). \end{aligned}$$

**(c)** Assume finally that $\mathscr {E}_{X'} \ne \emptyset$ and that no $x \in {\widehat{X}}$ has any proper descendant in *X*. This last condition implies that the set of nodes ${\widehat{X}} \setminus \mathscr {E}_X$ is independent and all their descendant exit reticulations belong to $\mathscr {E}_{X'}$. Then, by Lemma 2 we have that

7 $$\begin{aligned}&|{\widehat{X}} | = |{\widehat{X}} \setminus \mathscr {E}_X |+| \mathscr {E}_X| \leqslant (d-1) (k - l_X - l_{X'} - l_{X,X'}) + dl_{X'} +l_{X}\nonumber \\&\ = (d-1)k - (d-2)l_{X} + l_{X'} - (d-1)l_{X,X'} \nonumber \\&\ \leqslant (d-1)k +l_{X'} \qquad (\text {because} d\geqslant 2)\nonumber \\&\ \leqslant (d-1)k + \min \{k, |{\widehat{X}}' |\}\quad (\text {because} l_{X'} \leqslant k \text {and} l_{X'} \leqslant |{{\,\textrm{Supp}\,}}{\widehat{X}}' | \leqslant |{\widehat{X}}' |). \end{aligned}$$

In particular,

8 $$\begin{aligned} |{\widehat{X}} | \leqslant dk\text { and } |{\widehat{X}} | - |{\widehat{X}}' | \leqslant (d-1)k. \end{aligned}$$

Now, on the one hand, if $|{\widehat{X}} | < dk$, take $A = {\widehat{X}}$ and $B = {\widehat{X}}'$. By Eqns. (6) and (8), they satisfy the required conditions in the statement, and

$$\begin{aligned}&{{\,\textrm{Supp}\,}}\tau _{B,A}(X') = {{\,\textrm{Supp}\,}}\bigl ( (X' \setminus {\widehat{X}}') \cup {\widehat{X}} \bigr ) = {{\,\textrm{Supp}\,}}X, \\&{{\,\textrm{Supp}\,}}\tau _{A,B}(X) = {{\,\textrm{Supp}\,}}\bigl ( (X \setminus {\widehat{X}}) \cup {\widehat{X}}' \bigr ) = {{\,\textrm{Supp}\,}}X', \end{aligned}$$

which implies $${\uparrow }X'\cap {\uparrow }X={\uparrow }\tau _{A,B}(X)\cap {\uparrow }\tau _{B,A}(X')$$.

On the other hand, if $|{\widehat{X}} | = dk$, then all inequalities in the sequence (7) as well as the inequality $l_{X'} \leqslant k$ are equalities. The equality $l_{X'}=k$ implies that the blob ${\mathcal {B}}$ has no reticulation other than those in $\mathscr {E}_{X'}$. Moreover, since the first inequality in (7) is an equality, ${\widehat{X}}$ reaches the maximum number of possible independent nodes in ${\uparrow }\mathscr {E}_{X'}={\uparrow }\mathscr {E}$. Then, as noted in Remark 5, it must happen for each $H \in \mathscr {E}$ that $\deg _{in}(H) = d$ and $|{\widehat{X}} \cap {\uparrow }H | = |{\widehat{X}} \cap \uparrow _\textit{only}\, H | = d$.

Now, since $k = |\mathscr {E}_{X'} | \leqslant |{\widehat{X}}' | < |{\widehat{X}} | = dk$, there must exist some $H_0 \in \mathscr {E}_{X'}$ with $m_{X'}(H_0) < d$. Take $A = {\widehat{X}} \cap \uparrow _\textit{only}\, {H_0} = {\widehat{X}} \cap {\uparrow }H_0$ and *B* the multiset with ${{\,\textrm{Supp}\,}}B = \{H_0\}$ and $m_B(H_0) = m_{X'}(H_0)$. We have that $0<|B |<|A | = d$ and hence the pair (*A*, *B*) satisfies the requirements in the statement. As to Eqn. (5), notice that

$$\begin{aligned} {\uparrow }X'\subseteq&{\uparrow }(X'\setminus \{H_0\})\cup {\uparrow }A\cup \{H_0\}\\&\qquad \cup \{x'\in X'\mid x'\text { intermediate in some path }A\!\rightsquigarrow \! H_0\}. \end{aligned}$$

Now, $H_0\notin {\uparrow }X$ and, by assumption, the elements of *A* have no proper descendant in *X*, which implies

$$\begin{aligned} ( \{H_0\}\cup \{x'\in X'\mid x'\text { intermediate in some path }A\!\rightsquigarrow \! H_0\})\cap {\uparrow }X=\emptyset . \end{aligned}$$

Moreover, since $A\subseteq {\uparrow }H_0$, we have that ${\uparrow }X\subseteq {\uparrow }((X{\setminus } A)\cup \{H_0\})$. Therefore

$$\begin{aligned} {\uparrow }X'\cap {\uparrow }X\subseteq ({\uparrow }(X'\setminus \{H_0\})\cup {\uparrow }A)\cap {\uparrow }X\subseteq {\uparrow }((X'\setminus \{H_0\})\cup A)\cap {\uparrow }((X\setminus A)\cup \{H_0\}) \end{aligned}$$

as we wanted to prove. $\square$

### Theorem 1

If *N* is a semi-*d*-ary level-*k* phylogenetic network, $\textrm{rPSD}_N$ satisfies the exchange property with respect to $\mathscr {S}_{k,d}$.

### Proof

The case $k=0$ is Steel’s strong exchange property for phylogenetic trees (Steel 2016, §6.4.1). So, we shall focus on the case $k\geqslant 1$.

Without any loss of generality, we can assume that every tree node in *N* is at most bifurcating, in the sense that the out-degree of each tree node is at most 2 (recall that we do not forbid out-degree 1 tree nodes in our networks). Indeed, let first $N'$ be the phylogenetic network obtained from *N* as follows: for every node *v* that is the split node of more than one blob and for each such blob rooted at *v*, add a new split node $v_i$ to the blob and a new arc $(v,v_i)$ with weight 0. $N'$ is still semi-*d*-ary and level-*k*, no node in it is the split node of more than one blob, and $\textrm{rPSD}_N(Z)=\textrm{rPSD}_{N'}(Z)$ for every $Z\subseteq \Sigma$. Now, let $N''$ be the phylogenetic network obtained from $N'$ as follows: for every tree node *v* with $k\geqslant 3$ children $v_1,\ldots ,v_k$, replace in *N* the subgraph supported on $\{v,v_1,\ldots ,v_k\}$ by a bifurcating tree with root *v* and leaves $v_1,\ldots ,v_k$ and all its arcs except those ending in $v_1,\ldots ,v_k$ of weight 0: the arc ending in each $v_i$ inherits the original weight of $(v,v_i)$; if any node $v_i$ had any entering arcs other than $(v,v_i)$, we keep them with their weights. Since *v* was the split node of at most one blob, no blob increases its level from $N'$ to $N''$, and therefore $N''$ is still semi-*d*-ary and level-*k*, and $\textrm{rPSD}_{N''}(Z)=\textrm{rPSD}_{N'}(Z) =\textrm{rPSD}_{N}(Z)$ for every $Z\subseteq \Sigma$.

So, in the rest of this proof we shall suppose that *N* *is at-most-bifurcating* and in particular that no node in *N* is the split node of more than one blob.

We shall proceed by induction on the number $\alpha$ of arcs of the network. A phylogenetic network with $\alpha =0$ is a phylogenetic tree consisting of a single leaf, where the stated exchange property trivially holds. Now, let *N* be an at-most-bifurcating semi-*d*-ary level-*k* phylogenetic network with $\alpha \geqslant 1$ arcs, and let us suppose that the thesis in the statement is true for all at-most-bifurcating semi-*d*-ary level-*k* phylogenetic networks with less than $\alpha$ arcs.

Let $X, X'\subseteq \Sigma$ with $|X'| < |X|$. If $|X|=1$ the exchange property is trivially satisfied taking $A=X$ and $B=X'=\emptyset$, so we assume from now on that $|X|\geqslant 2$. Now consider the tree of blobs *T* of *N* (Gusfield et al. 2007), obtained by collapsing each blob in *N* into its split node. Then, *T* is a phylogenetic tree with the same root *r* as *N*, $V(T)\subseteq V(N)$, and, for every $v\in V(T)$, its cluster in *T* and in *N* are the same; let us denote it by *C*(*v*). Since $|X' | < |X |$ and $|X |\geqslant 2$, the set of nodes *v* in *T* such that $|X' \cap C(v) | < |X \cap C(v) |$ and $1<|X \cap C(v) |$ is nonempty: it contains the root *r*.

We shall consider four cases.

**(a)** Assume that *T* contains some node $v_0\ne r$ such that $|X\cap C(v_0)|>|X'\cap C(v_0)|$ and $|X\cap C(v_0)|>1$. Since $v_0\in V(T)$, $v_0$ is in *N* a tree node such that the arc $e_0=(v_1,v_0)$ ending in it does not belong to any blob, which implies that it is a cut arc. Let $N_0=N_{v_0}$ and let $N_1$ be the network obtained from *N* by removing $N_{v_0}$ and the arc $e_0$ and, if $v_1$ is a reticulation node, appending to it a dummy leaf child (not labelled in $\Sigma$) through an arc of weight 0; cf. Figure 6. By the induction hypothesis, $N_0$ satisfies the thesis in the statement.

Now, for every $Z\subseteq \Sigma$, if $Z \cap C(v_0) = \emptyset$, then $\textrm{rPSD}_N(Z)= \textrm{rPSD}_{N_1}(Z)$, and if $Z \cap C(v_0) \ne \emptyset$, then

$$\begin{aligned} \textrm{rPSD}_N(Z) = \textrm{rPSD}_{N_0}(Z) + \textrm{rPSD}_{N_1}(Z) + w(e_0) + \hspace{-3ex}\displaystyle \sum _{{e \in {\uparrow }v_1 \setminus {\uparrow }(Z \setminus C(v_0))}} \hspace{-3ex}w(e). \end{aligned}$$

(Throughout this proof, given a network $N'$ with set of leaves $\Sigma '$ and a set *Z*, we write $\textrm{rPSD}_{N'}(Z)$ to denote actually $\textrm{rPSD}_{N'}(Z\cap \Sigma ')$. So, for instance, $\textrm{rPSD}_{N_{0}}(Z)$ and $\textrm{rPSD}_{N_1}(Z)$ in the expressions above actually mean $\textrm{rPSD}_{N_{0}}(Z\cap C(v_0))$ and $\textrm{rPSD}_{N_{1}}(Z{\setminus } C(v_0))$, respectively.)

Since $|X\cap C(v_0)|>|X'\cap C(v_0)|$, by the induction hypothesis there exist $A\subseteq (X\setminus X')\cap C({v_{0}})$ and $B\subseteq (X'{\setminus } X)\cap C({v_{0}})$ such that $(A,B)\in \mathscr {S}_{k,d}(C(v_{0}))\subseteq \mathscr {S}_{k,d}$ and

9 $$\begin{aligned} \textrm{rPSD}_{N_0}(X) - \textrm{rPSD}_{N_0}(\tau _{A,B}(X)) \leqslant \textrm{rPSD}_{N_0}(\tau _{B,A}(X')) - \textrm{rPSD}_{N_0}(X'). \end{aligned}$$

Since $A, B \subseteq C(v_0)$, $\tau _{A,B}(X) {\setminus } C(v_0) = X {\setminus } C(v_0)$ and $\tau _{B,A}(X') {\setminus } C(v_0) = X' {\setminus } C(v_0)$, and thus, in particular,

$$\begin{aligned} \textrm{rPSD}_{N_1}(X) = \textrm{rPSD}_{N_1}(\tau _{A,B}(X)),\ \textrm{rPSD}_{N_1}(X') = \textrm{rPSD}_{N_1}(\tau _{B,A}(X')). \end{aligned}$$

Notice also that $\tau _{B,A}(X')\cap C(v_0)\ne \emptyset$ because $A\ne \emptyset$.

Assume first that $B \ne \emptyset$, so that $X' \cap C(v_0) \ne \emptyset$ and $\tau _{A,B}(X) \cap C(v_0) \ne \emptyset$. Then,

By the same argument, using that $X' \cap C(v_0) \ne \emptyset$ and $\tau _{B,A}(X')\cap C(v_0)\ne \emptyset$, we also have that

$$\begin{aligned}&\textrm{rPSD}_{N}(\tau _{B,A}(X')) - \textrm{rPSD}_{N}(X') = \textrm{rPSD}_{N_0}(\tau _{B,A}(X'))-\textrm{rPSD}_{N_0}(X'). \end{aligned}$$

Therefore, by Eqn. (9),

$$\begin{aligned}&\textrm{rPSD}_{N}(X) - \textrm{rPSD}_{N}(\tau _{A,B}(X))=\textrm{rPSD}_{N_0}(X) - \textrm{rPSD}_{N_0}(\tau _{A,B}(X))\\&\qquad \leqslant \textrm{rPSD}_{N_0}(\tau _{B,A}(X'))-\textrm{rPSD}_{N_0}(X')=\textrm{rPSD}_{N}(\tau _{B,A}(X')) - \textrm{rPSD}_{N}(X'). \end{aligned}$$

Assume now that $B = \emptyset$. Then, by the definition of $\mathscr {S}_{k,d}$, the set *A* must be a singleton and then $\tau _{A,B}(X) \cap C(v_0) =(X{\setminus } A)\cap C(v_0) \ne \emptyset$, because, by assumption, $|X \cap C(v_0) | > 1$. Then, arguing as above, we have that

$$\begin{aligned} \textrm{rPSD}_N(X) - \textrm{rPSD}_N(\tau _{A,B}(X)) = \textrm{rPSD}_{N_0}(X) - \textrm{rPSD}_{N_0}(\tau _{A,B}(X)). \end{aligned}$$

Similarly, if $$X' \cap C(v_0) \ne \emptyset $$,

$$\begin{aligned} \textrm{rPSD}_N(\tau _{B,A}(X')) - \textrm{rPSD}_N(X') = \textrm{rPSD}_{N_0}(\tau _{B,A}(X')) - \textrm{rPSD}_{N_0}(X'), \end{aligned}$$

while if $X' \cap C(v_0) = \emptyset$ (and using that $\tau _{B,A}(X') \setminus C(v_0)=X'\setminus C(v_0)$),

In either case, by Eqn. (9) we have again

$$\begin{aligned}&\textrm{rPSD}_{N}(X) - \textrm{rPSD}_{N}(\tau _{A,B}(X))=\textrm{rPSD}_{N_0}(X) - \textrm{rPSD}_{N_0}(\tau _{A,B}(X))\\&\qquad \leqslant \textrm{rPSD}_{N_0}(\tau _{B,A}(X'))-\textrm{rPSD}_{N_0}(X')\leqslant \textrm{rPSD}_{N}(\tau _{B,A}(X')) - \textrm{rPSD}_{N}(X'). \end{aligned}$$

**(b)** Assume now that the only node *v* in *T* such that $|X\cap C(v)|>|X'\cap C(v)|$ and $|X\cap C(v)|>1$ is the root *r*, and that *r* is not the split node of any blob in *N*. Then, each child *v* of *r* in *N* is also its child in *T* and thus, if $|X\cap C(v)|>|X'\cap C(v)|$, then $|X\cap C(v)|=1$. But since $|X|>|X'|$, *r* must have some child $v_1$ such that $|X\cap C(v_1)|>|X'\cap C(v_1)|$ and hence such that $|X\cap C(v_1)|=1$ and $X'\cap C(v_1)=\emptyset$; and then, since $|X|\geqslant 2$, *r* must have a second child $v_2$ and $X\cap C(v_2)\ne \emptyset$. For each $i=1,2$, let $e_i=(r,v_i)$ and let $N_{i}$ be the subnetwork of *N* rooted at $v_i$. The sets of leaves $C(v_1),C(v_2)$ of $N_1,N_2$ are disjoint and therefore, for each $Z\subseteq \Sigma$,

$$\begin{aligned} \textrm{rPSD}_N(Z) = \textrm{rPSD}_{N_{1}}(Z)+\textrm{rPSD}_{N_{2}}(Z)+\chi _{N_{1}}(Z)w(e_1)+\chi _{N_{2}}(Z)w(e_2) \end{aligned}$$

where, for each $i=1,2$, $\chi _{N_{i}}(Z) = 1$ if $Z\cap C({v_i})\ne \emptyset$ and $\chi _{N_{i}}(Z) = 0$ otherwise.

Let $X\cap C(v_1)=\{x\}$ and take $A=\{x\}$ and $B=\emptyset$. Then, $(A,B)\in \mathscr {S}_0$ and $\tau _{A,B}(X)\cap C(v_1)=\emptyset$, $\tau _{B,A}(X')\cap C(v_1)=\{x\}$, $\tau _{A,B}(X)\cap C(v_2)=X\cap C(v_2)$, and $\tau _{B,A}(X')\cap C(v_2)=X'\cap C(v_2)$. Therefore,

$$\begin{aligned}&\textrm{rPSD}_{N}(X) - \textrm{rPSD}_{N}(\tau _{A,B}(X))\\&\ = \textrm{rPSD}_{N_{1}}(X)+\textrm{rPSD}_{N_{2}}(X)+\chi _{N_{1}}(X)w(e_1)+\chi _{N_{2}}(X)w(e_2)- \textrm{rPSD}_{N_{1}}(\tau _{A,B}(X))\\&\qquad -\textrm{rPSD}_{N_{2}}(\tau _{A,B}(X))-\chi _{N_{1}}(\tau _{A,B}(X))w(e_1)-\chi _{N_{2}}(\tau _{A,B}(X))w(e_2) \\&\ = \textrm{rPSD}_{N_{1}}(\{x\}) + \textrm{rPSD}_{N_{2}}(X)+ w(e_1)+w(e_2) - 0 -\textrm{rPSD}_{N_{2}}(X) - 0 \cdot w(e_1) - w(e_2) \\&\ = \textrm{rPSD}_{N_{{1}}}(\{x\})+w(e_1) \end{aligned}$$

and, similarly,

$$\begin{aligned} \begin{array}{l} \textrm{rPSD}_N(\tau _{B,A}(X')) - \textrm{rPSD}_N(X')\\ \qquad = \textrm{rPSD}_{N_{1}}(\{x\}) + \textrm{rPSD}_{N_{2}}(X')+ w(e_1)+\chi _{N_{2}}(X')w(e_2)\\ \qquad \qquad - 0 -\textrm{rPSD}_{N_{2}}(X') - 0 \cdot w(e_1) - \chi _{N_{2}}(X')w(e_2) \\ \qquad = \textrm{rPSD}_{N_{{1}}}(\{x\}) +w(e_1). \end{array} \end{aligned}$$

Hence, in this case,

$$\begin{aligned} \textrm{rPSD}_{N}(X) - \textrm{rPSD}_{N}(\tau _{A,B}(X))= \textrm{rPSD}_N(\tau _{B,A}(X')) - \textrm{rPSD}_N(X'). \end{aligned}$$

**(c)** Assume finally that the only node *v* in *T* such that $|X\cap C(v)|>|X'\cap C(v)|$ and $|X\cap C(v)|>1$ is the root *r*, and that *r* is the split node of a (single) blob ${\mathcal {B}}$. we distinguish two subcases.

**(c.1)** If ${\mathcal {B}}$ contains some exit reticulation *H* with no descendant in $X\cup X'$, and if $v_1,\ldots ,v_{d'}$ are the parents of *H*, then let $\widehat{N}$ be the phylogenetic network obtained from *N* by removing the subnetwork ${N}_{H}$, adding new leaves $h_1,\ldots ,h_{d'}$ with dummy labels outside $\Sigma$, and replacing each arc $(v_i,H)$ by an arc $(v_i,h_i)$ with weight 0; cf. Figure 7. $\widehat{N}$ is still at-most-bifurcating, semi-*d*-ary, and level-*k* and it has less than $\alpha$ arcs (we have removed the arcs in $N_H$). Therefore, by the induction hypothesis, it satisfies the thesis in the statement. Let ${\widehat{\Sigma }}$ be its set of labels. Then, since, by assumption, $X,X'\subseteq {\widehat{\Sigma }}\cap \Sigma$, there exist $A \subseteq X{\setminus } X'$ and $B \subseteq X' {\setminus } X$ such that $(A,B)\in \mathscr {S}_{k,d}({\widehat{\Sigma }}\cap \Sigma )\subseteq \mathscr {S}_{k,d}$ and

$$\begin{aligned} \textrm{rPSD}_{\widehat{N}}(X) - \textrm{rPSD}_{\widehat{N}}(\tau _{A,B}(X)) \leqslant \textrm{rPSD}_{\widehat{N}}(\tau _{B,A}(X')) - \textrm{rPSD}_{\widehat{N}}(X'). \end{aligned}$$

Since $\textrm{rPSD}_{\widehat{N}}(Z)= \textrm{rPSD}_{N}(Z)$ for every $Z\subseteq {\widehat{\Sigma }}\cap \Sigma$, we conclude that

$$\begin{aligned} \textrm{rPSD}_{N}(X) - \textrm{rPSD}_{N}(\tau _{A,B}(X)) \leqslant \textrm{rPSD}_{N}(\tau _{B,A}(X')) - \textrm{rPSD}_{N}(X'). \end{aligned}$$

**(c.2)** Finally, assume that all the exit reticulations of the blob ${\mathcal {B}}$ rooted at *r* have descendants in *X* or $X'$. Let ${\mathcal {B}}^*$ be the set of nodes of ${\mathcal {B}}$ that have a child outside of ${\mathcal {B}}$; if $v\in {\mathcal {B}}^*$, we shall denote its child outside of ${\mathcal {B}}$ by ${\bar{v}}$. Notice that:

- $r\notin {\mathcal {B}}^*$ (its two children must belong to the blob);
- the exit reticulations of ${\mathcal {B}}$ belong to ${\mathcal {B}}^*$;
- since reticulations have out-degree 1, the internal reticulations of ${\mathcal {B}}$ do not belong to ${\mathcal {B}}^*$;
- ${\bar{v}}\in V(T){\setminus }\{r\}$ for every $v\in {\mathcal {B}}^*$, and thus, by the current assumption, if $|X\cap C({\bar{v}})|>|X'\cap C({\bar{v}})|$ then $|X\cap C({\bar{v}})|=1$.

For each $v \in {\mathcal {B}}^*$ let ${\overline{N}}_v$ be the subnetwork of *N* rooted at *v* consisting of $N_{{\bar{v}}}$, *v* and the arc $(v, {\bar{v}})$.

For each $Z \subseteq \Sigma$, we shall denote by ${\mathcal {B}}_Z^*$ the multiset of nodes of ${\mathcal {B}}^*$ supported on

$$\begin{aligned} {{\,\textrm{Supp}\,}}{\mathcal {B}}_Z^*=\{v\in {\mathcal {B}}^*:\, Z \cap C({\bar{v}})\ne \emptyset \} \end{aligned}$$

and with multiplicities $m_{{\mathcal {B}}_Z^*}(v) = |Z \cap C(\bar{v}) |$. Since the subnetworks ${\overline{N}}_v$, with $v \in \mathcal B^*$, have pairwise disjoint sets of leaves and the union of their sets of leaves is $\Sigma$, we have that $|{\mathcal {B}}_Z^*|=|Z|$ and

10 $$\begin{aligned} \textrm{rPSD}_N(Z) = \sum _{v \in {{\,\textrm{Supp}\,}}{\mathcal {B}}_Z^*} \textrm{rPSD}_{{\overline{N}}_v}(Z) + \sum _{e \in {\uparrow }{\mathcal {B}}_Z^* } w(e). \end{aligned}$$

So, $|{\mathcal {B}}_{X'}^*|=|X'|<|X|=|{\mathcal {B}}_X^*|$; by the current assumption, every exit reticulation belongs to ${\mathcal {B}}_X^*\cup {\mathcal {B}}_{X'}^*$; and if $m_{{\mathcal {B}}_{X'}^*}(v)=|X'\cap C(\bar{v})|<m_{{\mathcal {B}}_X^*}(v)=|X\cap C({\bar{v}})|$, then $m_{\mathcal B_X^*}(v)=1$. Therefore, the multisets ${\mathcal {B}}_X^*$, $\mathcal B_{X'}^*$ satisfy the hypotheses of Lemma 3, which implies the existence of a set ${\mathcal {B}}_A$ and a multiset ${\mathcal {B}}_B$ of nodes of ${\mathcal {B}}$ such that:

1. ${\mathcal {B}}_A \subseteq {{\,\textrm{Supp}\,}}{\mathcal {B}}_X^* {\setminus } {{\,\textrm{Supp}\,}}{\mathcal {B}}_{X'}^*$; thus, if $v\in {\mathcal {B}}_A$, $|X\cap C({\bar{v}})|=1$ and $|X'\cap C({\bar{v}})|=0$.
2. ${{\,\textrm{Supp}\,}}{\mathcal {B}}_B \subseteq {{\,\textrm{Supp}\,}}{\mathcal {B}}_{X'}^* {\setminus } {{\,\textrm{Supp}\,}}{\mathcal {B}}_X^*$ and, for every $v\in {{\,\textrm{Supp}\,}}\mathcal B_B$, $m_{{\mathcal {B}}_B}(v)=m_{{\mathcal {B}}^*_{X'}}(v)=|X'\cap C({\bar{v}})|$.
3. ${\mathcal {B}}_B=\emptyset$ and $|{\mathcal {B}}_A | = 1$, or $0< |{\mathcal {B}}_B | < |{\mathcal {B}}_A |= d$, or $0< |{\mathcal {B}}_B |< |{\mathcal {B}}_A | < dk$ and $|\mathcal B_A | - |{\mathcal {B}}_B | \leqslant (d-1)k$.
4. $${\uparrow }{\mathcal {B}}_X^*\cap {\uparrow }{\mathcal {B}}_{X'}^*\subseteq {\uparrow }\tau _{{\mathcal {B}}_A,{\mathcal {B}}_B}({\mathcal {B}}_{X}^*)\cap {\uparrow }\tau _{{\mathcal {B}}_B,{\mathcal {B}}_A}({\mathcal {B}}_{X'}^*)$$.

Let

$$\begin{aligned} A = \bigcup _{v \in {\mathcal {B}}_A} (X \cap C({\bar{v}})),\quad B = \bigcup _{v \in {{\,\textrm{Supp}\,}}{\mathcal {B}}_B} (X' \cap C({\bar{v}})). \end{aligned}$$

Then, $A\subseteq X\setminus X'$ and $B\subseteq X'\setminus X$ with $|A | = |{\mathcal {B}}_A |$ and $|B | = |{\mathcal {B}}_B |$. In particular, by property (3), $(A,B) \in \mathscr {S}_{k,d}$. We shall prove that

$$\begin{aligned} \textrm{rPSD}_N (X) - \textrm{rPSD}_N(\tau _{A,B}(X))\leqslant \textrm{rPSD}_N( \tau _{B,A}(X')) - \textrm{rPSD}_N(X'). \end{aligned}$$

Before doing so, let us point out some facts that we shall use. First, notice that ${\mathcal {B}}_A={\mathcal {B}}_A^*$ and $\mathcal B_B={\mathcal {B}}_B^*$, because for every $v\in {\mathcal {B}}^*$

$$\begin{aligned}&m_{{\mathcal {B}}_A^*}(v) =|A\cap C({\bar{v}})|=\left\{ \begin{array}{l} 1 \text { if}\, v\in A\\ 0 \text { if}\, v\notin A \end{array}\right\} =m_{{\mathcal {B}}_A}(v)\\&m_{{\mathcal {B}}_B^*}(v) =|B\cap C({\bar{v}})|=|X'\cap C({\bar{v}})|\\&\qquad (\text {because the clusters} C({\bar{v}}) \text {are pairwise disjoint})\\&\qquad =m_{{\mathcal {B}}_{X'}^*}(v)=m_{{\mathcal {B}}_B}(v). \qquad \text {(by definition)} \end{aligned}$$

Moreover

11 $$\begin{aligned}&{\mathcal {B}}_{\tau _{A,B}(X)}^*=\tau _{{\mathcal {B}}_A,\mathcal B_B}({\mathcal {B}}_{X}^*) \text { and } {{\,\textrm{Supp}\,}}\mathcal B_{\tau _{A,B}(X)}^*=( ({{\,\textrm{Supp}\,}}{\mathcal {B}}_X^*) \setminus {\mathcal {B}}_A) \cup {{\,\textrm{Supp}\,}}{\mathcal {B}}_B, \end{aligned}$$

12 $$\begin{aligned}&{\mathcal {B}}_{\tau _{B,A}(X')}^*=\tau _{{\mathcal {B}}_B,\mathcal B_A}({\mathcal {B}}_{X'}^*) \text { and } {{\,\textrm{Supp}\,}}\mathcal B_{\tau _{B,A}(X')}^*= ( {{\,\textrm{Supp}\,}}{\mathcal {B}}_{X'}^* \setminus {{\,\textrm{Supp}\,}}{\mathcal {B}}_B) \cup {\mathcal {B}}_A. \end{aligned}$$

Indeed, as to Eqn. (11), for every $v\in {\mathcal {B}}^*$

$$\begin{aligned}&m_{{\mathcal {B}}_{\tau _{A,B}(X)}^*}(v)=|((X\setminus A)\cup B)\cap C({\bar{v}})|= |X\cap C({\bar{v}})|-|A\cap C({\bar{v}})|+ |B\cap C({\bar{v}})|\\&\quad = m_{{\mathcal {B}}_{X}^*}(v)- m_{{\mathcal {B}}_{A}}(v)+m_{\mathcal B_B}(v)=m_{{\mathcal {B}}_{X}^*\setminus {\mathcal {B}}_{A}}(v)+m_{\mathcal B_B}(v) = m_{({\mathcal {B}}_{X}^*\setminus {\mathcal {B}}_{A})\cup \mathcal B_B}(v) \end{aligned}$$

and in particular

$$\begin{aligned} {{\,\textrm{Supp}\,}}{\mathcal {B}}_{\tau _{A,B}(X)}^*={{\,\textrm{Supp}\,}}(({\mathcal {B}}_X^*\setminus {\mathcal {B}}_A)\cup {\mathcal {B}}_B)= ( ({{\,\textrm{Supp}\,}}{\mathcal {B}}_X^*) \setminus {\mathcal {B}}_A) \cup {{\,\textrm{Supp}\,}}{\mathcal {B}}_B^* \end{aligned}$$

because $m_{{\mathcal {B}}_{A}}(v)=m_{{\mathcal {B}}_{X}^*}(v)$ for every $v\in {\mathcal {B}}_A$.

A similar argument, using that, for every $v\in {{\,\textrm{Supp}\,}}\mathcal B_B$, $m_{{\mathcal {B}}_{B}}(v)=m_{{\mathcal {B}}_{X'}^*}(v)=|B\cap C(\bar{v})|=|X'\cap C({\bar{v}})|$ and that ${\mathcal {B}}_A\cap {{\,\textrm{Supp}\,}}\mathcal B_{X'}^*=\emptyset$, proves Eqn. (12).

We can proceed now to prove the desired inequality

$$\begin{aligned} \textrm{rPSD}_N (X) - \textrm{rPSD}_N(\tau _{A,B}(X))\leqslant \textrm{rPSD}_N( \tau _{B,A}(X')) - \textrm{rPSD}_N(X'). \end{aligned}$$

By Eqn. (10),

13 $$\begin{aligned}&\textrm{rPSD}_N (X) - \textrm{rPSD}_N(\tau _{A,B}(X)) \nonumber \\&\quad = \hspace{-2ex}\sum _{v \in {{\,\textrm{Supp}\,}}\mathcal B_X^*}\hspace{-2ex} \textrm{rPSD}_{{\overline{N}}_v}(X) - \hspace{-4ex}\sum _{{v \in {{\,\textrm{Supp}\,}}{\mathcal {B}}_{\tau _{A,B}(X)}^*}} \hspace{-4ex} \textrm{rPSD}_{{\overline{N}}_v}(\tau _{A,B}(X))+ \sum _{e \in {\uparrow }{\mathcal {B}}_X^* } w(e) - \hspace{-4ex}\sum _{e \in {\uparrow }{\mathcal {B}}_{\tau _{A,B}(X)}^* }\hspace{-4ex} w(e) \end{aligned}$$

where

14 $$\begin{aligned}&\sum _{v \in {{\,\textrm{Supp}\,}}{\mathcal {B}}_X^*} \textrm{rPSD}_{{\overline{N}}_v}(X) = \hspace{-4ex}\sum _{v \in ( {{\,\textrm{Supp}\,}}{\mathcal {B}}_X^*) \setminus \mathcal B_A}\hspace{-4ex} \textrm{rPSD}_{{\overline{N}}_v}(X) + \sum _{v \in {\mathcal {B}}_A} \textrm{rPSD}_{{\overline{N}}_v}(X) \nonumber \\&\qquad = \sum _{v \in ({{\,\textrm{Supp}\,}}{\mathcal {B}}_X^*) \setminus \mathcal B_A}\hspace{-2ex} \textrm{rPSD}_{{\overline{N}}_v}((X \setminus A)) + \sum _{v \in {\mathcal {B}}_A} \textrm{rPSD}_{{\overline{N}}_v}(A) \end{aligned}$$

because if $v \in ({{\,\textrm{Supp}\,}}{\mathcal {B}}_X^*) \setminus {\mathcal {B}}_A$, then $A \cap C({\bar{v}}) = \emptyset$ and if $v \in {\mathcal {B}}_A$, then $X \cap C({\bar{v}})=A \cap C({\bar{v}})$; and

15 $$\begin{aligned}&\sum _{{v \in {{\,\textrm{Supp}\,}}{\mathcal {B}}_{\tau _{A,B}(X)}^*}}\hspace{-3ex} \textrm{rPSD}_{{\overline{N}}_v}(\tau _{A,B}(X)) \nonumber \\&\qquad \qquad =\hspace{-5ex} \sum _{{v \in ({{\,\textrm{Supp}\,}}{\mathcal {B}}_X^*) \setminus {\mathcal {B}}_A}}\hspace{-5ex} \textrm{rPSD}_{{\overline{N}}_v}\bigl (((X \setminus A) \cup B)\bigr )+\hspace{-2ex} \sum _{{v \in {{\,\textrm{Supp}\,}}{\mathcal {B}}_B}} \hspace{-2ex} \textrm{rPSD}_{{\overline{N}}_v}\bigl (((X \setminus A) \cup B)\bigr ) \nonumber \\&\qquad (\text {by} 11) \nonumber \\&\qquad \qquad = \hspace{-5ex}\sum _{{v \in ({{\,\textrm{Supp}\,}}{\mathcal {B}}_X^*) \setminus {\mathcal {B}}_A}}\hspace{-4ex} \textrm{rPSD}_{{\overline{N}}_v}((X \setminus A)) + \hspace{-2ex}\sum _{v \in {{\,\textrm{Supp}\,}}\mathcal B_B}\hspace{-2ex} \textrm{rPSD}_{{\overline{N}}_v}(B) \end{aligned}$$

because if $v \in {{\,\textrm{Supp}\,}}{\mathcal {B}}_X^*$, then $B\cap C(\bar{v})=\emptyset$, and if $v \in {{\,\textrm{Supp}\,}}{\mathcal {B}}_B$, then $X \cap C({\bar{v}}) = \emptyset$.

Therefore, combining Eqns. (13) to (15), we obtain

$$\begin{aligned}&\textrm{rPSD}_N( X) - \textrm{rPSD}_N(\tau _{A,B}(X)) = \\&\quad = \sum _{v \in {\mathcal {B}}_A} \textrm{rPSD}_{{\overline{N}}_v}(A) - \sum _{v \in {{\,\textrm{Supp}\,}}{\mathcal {B}}_B}\hspace{-2ex} \textrm{rPSD}_{{\overline{N}}_v}(B) +\hspace{-1ex} \sum _{e \in {\uparrow }{\mathcal {B}}_X^* }\hspace{-1ex} w(e) - \hspace{-2ex}\sum _{e \in {\uparrow }{\mathcal {B}}_{\tau _{A,B}(X)}^* }\hspace{-3ex} w(e). \end{aligned}$$

A similar argument proves that

$$\begin{aligned}&\textrm{rPSD}_N( \tau _{B,A}(X')) - \textrm{rPSD}_N(X') \\&\quad = \sum _{v \in {\mathcal {B}}_A} \textrm{rPSD}_{{\overline{N}}_v}(A) - \sum _{v \in \mathcal {{\,\textrm{Supp}\,}}B_B} \textrm{rPSD}_{{\overline{N}}_v}(B) + \hspace{-2ex}\sum _{e \in {\uparrow }{\mathcal {B}}_{\tau _{B,A}(X')}^* }\hspace{-3ex} w(e)-\hspace{-1ex} \sum _{e \in {\uparrow }\mathcal B_{X'}^* }\hspace{-1ex} w(e). \end{aligned}$$

Thus,

$$\begin{aligned} \textrm{rPSD}_N (X) - \textrm{rPSD}_N(\tau _{A,B}(X))\leqslant \textrm{rPSD}_N( \tau _{B,A}(X')) - \textrm{rPSD}_N(X') \end{aligned}$$

if, and only if,

$$\begin{aligned} \hspace{-1ex} \sum _{e \in {\uparrow }{\mathcal {B}}_X^* }\hspace{-1ex} w(e) +\hspace{-1ex} \sum _{e \in {\uparrow }{\mathcal {B}}_{X'}^* }\hspace{-1ex} w(e) \leqslant \hspace{-2ex}\sum _{e \in {\uparrow }\mathcal B_{\tau _{A,B}(X)}^* }\hspace{-3ex} w(e) + \hspace{-2ex}\sum _{e \in {\uparrow }{\mathcal {B}}_{\tau _{B,A}(X')}^* }\hspace{-3ex} w(e). \end{aligned}$$

Finally, this last inequality holds because

$$\begin{aligned}&\sum _{e \in {\uparrow }{\mathcal {B}}_X^*} w(e)+ \sum _{e \in {\uparrow }{\mathcal {B}}_{X'}^*} w(e) =\sum _{e \in {\uparrow }{\mathcal {B}}_X^*\cup {\uparrow }{\mathcal {B}}_{X'}^*} w(e)+ \sum _{e \in {\uparrow }{\mathcal {B}}_X^*\cap {\uparrow }{\mathcal {B}}_{X'}^*} w(e) \\&\quad \leqslant \sum _{e \in {\uparrow }{\mathcal {B}}_{\tau _{A,B}(X)}^* \cup {\uparrow }{\mathcal {B}}_{\tau _{B,A}(X')}^*} w(e)+ \sum _{e \in {\uparrow }{\mathcal {B}}_{\tau _{A,B}(X)}^* \cap {\uparrow }{\mathcal {B}}_{\tau _{B,A}(X')}^*} w(e)\qquad \qquad (*)\\&\quad = \sum _{e \in {\uparrow }\mathcal B_{\tau _{A,B}(X)}^*}w(e)+\sum _{e \in {\uparrow }\mathcal B_{\tau _{B,A}(X')}^*} w(e) \end{aligned}$$

where step $(*)$ is due to

$$\begin{aligned}&{\uparrow }{\mathcal {B}}_{\tau _{A,B}(X)}^* \cup {\uparrow }\mathcal B_{\tau _{B,A}(X')}^* ={\uparrow }({\mathcal {B}}_{\tau _{A,B}(X)}^* \cup {\mathcal {B}}_{\tau _{B,A}(X')}^*)\\&\quad = {\uparrow }(\tau _{{\mathcal {B}}_A,{\mathcal {B}}_B} ({\mathcal {B}}_X^*) \cup \tau _{{\mathcal {B}}_B,{\mathcal {B}}_A} ({\mathcal {B}}_{X'}^*)) \qquad (\text {by} (11) \text {and} (12))\\&\quad = {\uparrow }\big (((({\mathcal {B}}_X^*\setminus {\mathcal {B}}_A)\cup {\mathcal {B}}_B) \cup (({\mathcal {B}}_{X'}^*\setminus {\mathcal {B}}_B)\cup {\mathcal {B}}_A))\big )\\&\quad ={\uparrow }({\mathcal {B}}_X^* \cup {\mathcal {B}}_{X'}^*)={\uparrow }{\mathcal {B}}_X^* \cup {\uparrow }{\mathcal {B}}_{X'}^* \end{aligned}$$

and, by property (4) of ${\mathcal {B}}_A$ and ${\mathcal {B}}_B$ (and, again, (11) and (12)),

$$\begin{aligned} {\uparrow }{\mathcal {B}}_X^* \cap {\uparrow }{\mathcal {B}}_{X'}^*\subseteq {\uparrow }\tau _{{\mathcal {B}}_A,{\mathcal {B}}_B} ({\mathcal {B}}_X^*) \cap {\uparrow }\tau _{{\mathcal {B}}_B,{\mathcal {B}}_A} ({\mathcal {B}}_{X'}^*)= {\uparrow }{\mathcal {B}}_{\tau _{A,B}(X)}^* \cup {\uparrow }\mathcal B_{\tau _{B,A}(X')}^*. \end{aligned}$$

This completes the proof of case **(c.2)**. $\square$

## Footnotes

1 A class of undirected graphs that generalize unrooted trees and do not describe evolutionary histories but simply evolutionary relationships.

2 A subclass of split networks widely used because they are the output of popular programs like PhyloNet (Yu et al. 2014) or Splitstree4 (Huson and Bryant 2006).
