# Supplementary material for: An interchange property for the rooted phylogenetic subnet diversity on phylogenetic networks
Source: J Math Biol. 2024 Oct 4;89(5):48. doi: 10.1007/s00285-024-02142-4 (PMC11452452; doi:10.1007/s00285-024-02142-4)
Supplement: Supplementary file 1 — (pdf 487 KB) [file 285_2024_2142_MOESM1_ESM.pdf]

An interchange property for the rooted Phylogenetic Subnet Diversity on phylogenetic networks. Supplementary Material

Tomás M. Coronado[1], Gabriel Riera[1], Francesc Rosselló[1]

[1]Dept. of Mathematics and Computer Science, University of the Balearic Islands, Palma, E-07122, Spain, and Balearic Islands Health Research Institute (IdISBa), Palma, E-07010, Spain

{t.martinez,gabriel.riera,cesc.rossello}@uib.eu.

1 Proof of Lemma 9

Given a blob $\mathcal{B}$ with set of exit reticulations $\mathscr{E}$ and a subset $\mathscr{E}_1 \subseteq \mathscr{E}$, we denote by $\Uparrow_{\mathcal{B},only} \mathscr{E}_1$ the set of nodes of $\mathcal{B}$ with all their descendant exit reticulations in $\mathscr{E}_1$:

$$\Uparrow_{\mathcal{B},only} \mathscr{E}_1 = \Uparrow\mathscr{E}_1 \setminus \Uparrow(\mathscr{E} \setminus \mathscr{E}_1) = V(\mathcal{B}) \setminus \Uparrow(\mathscr{E} \setminus \mathscr{E}_1).$$

This notation extends the notation $\Uparrow_{only} H$ for $H \in \mathscr{E}$ used in the proof of Lemma 10.

We shall actually prove a slightly more general result than Lemma 9:

Lemma 11. *Let $\mathcal{B}$ be a blob, let $\mathscr{E}_1$ be a subset of its exit reticulations, and let $\mathcal{I}_1$ be the set of its internal reticulations belonging to $\Uparrow_{\mathcal{B},only} (\mathscr{E}_1)$. Then, for every independent set of nodes V contained in $\Uparrow_{\mathcal{B},only} (\mathscr{E}_1)$,*

$$|V| \leqslant \sum_{H_i \in \mathscr{E}_1} \deg_{in} H_i + \sum_{H_i \in \mathcal{I}_1} (deg_{in} H_i - 1).$$

Proof. Let $\mathcal{I}$ and $\mathscr{E}$ be the sets of internal and exit reticulations, respectively, of the blob $\mathcal{B}$. Let $\mathscr{E}_1 = \{H_1, \ldots, H_{l_1}\} \subseteq \mathscr{E}$ and $\mathcal{I}_1 = \mathcal{I} \cap \Uparrow_{\mathcal{B},only} (\mathscr{E}_1) = \{H_{l_1+1}, \ldots, H_{l_1+k_1}\}$. For each $i = 1, \ldots, l_1+k_1$, let $d_i = \deg_{in} H_i$. We shall prove that, for every independent

subset V of $\upharpoonleft_{\mathcal{B},only}\mathscr{E}_1$,

$$|V| \leqslant \sum_{i=1}^{l_1} d_i + \sum_{i=l_1+1}^{l_1+k_1} (d_i - 1)$$

by double induction on $l_1 = |\mathscr{E}_1|$ and $k_{int} = |\mathcal{I}|$. The case when $l_1 = 0$ is obvious, because then $\upharpoonleft_{\mathcal{B},only}(\mathscr{E}_1) = \emptyset$ and hence $|V| = 0$.

To prove the general inductive step from $l_1 - 1$ to l_1, we begin with the case when $k_{int} = 0$. So, let $\mathcal{B}$ be a blob without internal reticulations, let $\mathscr{E}$ be its set of exit reticulations, and let $\mathscr{E}_1 = \{H_1, \ldots, H_{l_1}\} \subseteq \mathscr{E}$ with $l_1 \geqslant 1$. Let us assume, as induction hypothesis, that the thesis is true for all blobs $\mathcal{B}'$ without internal reticulations and for all subsets of exit reticulations $\mathscr{E}_1'$ of $\mathcal{B}'$ of cardinality $|\mathscr{E}_1'| = l_1 - 1$.

Take a node $H_{l_1} \in \mathscr{E}_1$, with parents $u_1, \ldots, u_{d_{l_1}}$. For each $i = 1, \ldots, d_{l_1}$, let v_i be the lowest ancestor of u_i that has some descendant (exit) reticulation other than H_{l_1}; see Figure 9. Concatenating each path $v_i \rightsquigarrow u_i$ with the corresponding arc (u_i, H_{l_1}), we obtain d_{l_1} different paths $v_1 \rightsquigarrow H_{l_1}, \ldots, v_{d_{l_1}} \rightsquigarrow H_{l_1}$ ending in H_{l_1}: observe that the nodes $v_1, \ldots, v_{d_{l_1}}$ need not be different, but each such path ends in a different arc (u_i, H_{l_1}).

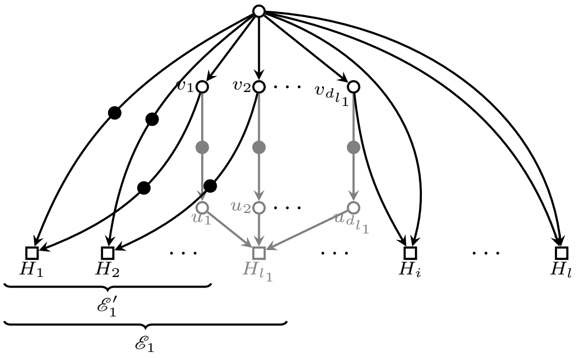

Fig. 8 A semibinary blob $\mathcal{B}$ without internal reticulations illustrating the inductive step for $k_{int} = 0$ in the proof of Lemma 11. All arcs in it except those ending in H_{l_1} actually represent paths. An independent set of nodes in $\upharpoonleft_{\mathcal{B},only}(\mathscr{E}_1)$ is represented by filled circles, and the nodes and arcs that are removed from $\mathcal{B}$ to $\mathcal{B}'$ are represented in gray.

Let $\mathcal{B}'$ be the directed graph obtained by removing from $\mathcal{B}$ the set of nodes $\upharpoonleft_{\mathcal{B},only} H_{l_1}$ —that is, the reticulation H_{l_1} and the intermediate nodes of the paths $v_1 \rightsquigarrow H_{l_1}, \ldots, v_{d_{l_1}} \rightsquigarrow H_{l_1}$— together with the arcs incident to them. The set of exit reticulations of $\mathcal{B}'$ is $\mathscr{E}' = \mathscr{E} \setminus \{H_{l_1}\}$; take $\mathscr{E}_1' = \{H_1, \ldots, H_{l_1-1}\}$. Since $\mathcal{B}$ did not have internal reticulations, neither does $\mathcal{B}'$. Then, by the induction hypothesis, $|V'| \leqslant \sum_{i=1}^{l_1-1} d_i$ for every independent set $V' \subseteq \upharpoonleft_{\mathcal{B}',only}(\mathscr{E}_1')$.

Now, since $V(\mathcal{B}) = V(\mathcal{B}') \sqcup \upharpoonleft_{\mathcal{B},only} H_{l_1}$, we have that

$$\upharpoonleft_{\mathcal{B},only}(\mathscr{E}_1) = \upharpoonleft_{\mathcal{B}',only}(\mathscr{E}_1') \sqcup \upharpoonleft_{\mathcal{B},only} H_{l_1}.$$

Therefore, any independent subset V' of $\Uparrow_{\mathcal{B}',only}(\mathscr{E}_1')$ can be enlarged to an independent subset V of $\Uparrow_{\mathcal{B},only}(\mathscr{E}_1)$ by adding at most d_{l_1} nodes, one inside each path $v_i \rightsquigarrow H_{l_1}$. Conversely, if we remove from an independent set of nodes V in $\mathcal{B}$ its nodes that are intermediate in the paths $v_1 \rightsquigarrow H_{l_1}, \ldots, v_{d_{l_1}} \rightsquigarrow H_{l_1}$ (and by the independence condition each such path will contain at most one element of V and therefore we remove in this way at most d_{l_1} nodes), or the node H_{l_1} if it belongs to V (and then no intermediate node in any path $v_i \rightsquigarrow H_{l_1}$ will belong to V), we obtain an independent subset V' of $\mathcal{B}'$.

Then, since $|V'| \leqslant \sum_{i=1}^{l_1-1} d_i$, any independent subset of $\Uparrow_{\mathcal{B},only}(\mathscr{E}_1)$ has cardinality at most $\sum_{i=1}^{l_1-1} d_i + d_{l_1}$. This proves this inductive step when $k_{int} = 0$.

Let us prove now, for any fixed $l_1 > 0$, the inductive step from $k_{int} - 1$ to k_{int}. So, assume that the thesis in the statement is true for all blobs $\mathcal{B}'$ with $k_{int} - 1$ internal reticulations and subsets $\mathscr{E}_1'$ of exit reticulations of cardinality $|\mathscr{E}_1'| \leqslant l_1$, and let $\mathcal{B}$ be a blob with k_{int} internal reticulations and $\mathscr{E}_1 = \{H_1, \ldots, H_{l_1}\}$ a set of $l_1 \geqslant 1$ exit reticulations. Let $\mathcal{I}_1 = \mathcal{I} \cap \Uparrow_{\mathcal{B},only}(\mathscr{E}_1) = \{H_{l_1+1}, \ldots, H_{l_1+k_1}\}$.

Let H be an internal reticulation with no reticulate proper ancestor and let $u_1, \ldots, u_d$ be its parents. For each $i = 1, \ldots, d$, let v_i be the lowest ancestor of u_i with some path to an exit reticulation that does not contain H. Concatenating each path $v_i \rightsquigarrow u_i$ with the corresponding arc (u_i, H), we obtain d different paths $v_1 \rightsquigarrow H, \ldots, v_d \rightsquigarrow H$ ending in H: as before, observe that the nodes $v_1, \ldots, v_d$ need not be different, but each such path ends in a different arc (u_i, H).

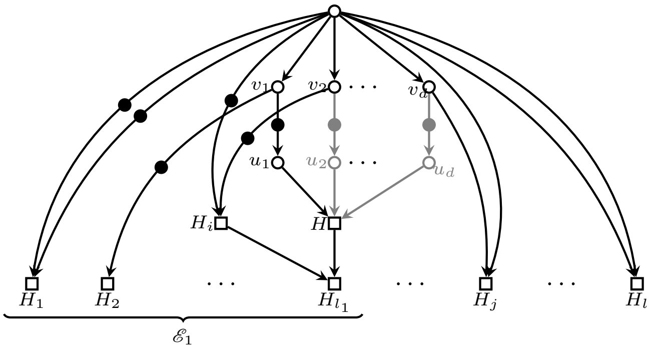

Fig. 9 A semibinary blob $\mathcal{B}$ illustrating the inductive step from $k_{int} - 1$ to k_{int} in the proof of Lemma 11. All arcs in it except those ending in H actually represent paths. An independent set of nodes in $\Uparrow_{\mathcal{B},only}(\mathscr{E}_1)$ is represented by filled circles. The nodes and arcs removed from N to N' are represented in gray.

Let $\mathcal{B}'$ be the directed graph obtained by removing the intermediate nodes in all paths $v_i \rightsquigarrow H$ except for one path $v_1 \rightsquigarrow H$, together with the arcs incident to them; if $v_i = u_i$, we simply remove the arc (u_i, H). The blob $\mathcal{B}'$ still has the same set of exit reticulations $\mathscr{E}$ as $\mathcal{B}$, but it has $k_{int} - 1$ internal reticulations because H has become an elementary tree node in $\mathcal{B}'$. Moreover, if we denote by $\mathcal{I}_1'$ the set of internal reticulations in $\Uparrow_{\mathcal{B}',only}(\mathscr{E}_1)$, then $\mathcal{I}_1' = \mathcal{I}_1$ if $H \notin \mathcal{I}_1$ and $\mathcal{I}_1' = \mathcal{I}_1 \setminus \{H\}$ if $H \in \mathcal{I}_1$. If

this last case happens, let us assume without any loss of generality that $H = H_{l_1+k_1}$ and hence that $d = d_{l_1+k_1}$.

Then, by the induction hypothesis, for every independent set $V' \subseteq \uparrow_{\mathcal{B}',only}(\mathscr{E}_1)$

$$|V'| \leqslant \begin{cases} \sum_{i=1}^{l_1} d_1 + \sum_{i=l_1+1}^{l_1+k_1}(d_i - 1) & \text{if } H \notin \mathcal{I}_1 \\ \sum_{i=1}^{l_1} d_1 + \sum_{i=l_1+1}^{l_1+k_1-1}(d_i - 1) & \text{if } H = H_{l_1+k_1} \in \mathcal{I}_1 \end{cases} \quad (16)$$

Now, notice that any independent set of nodes V' in $\mathcal{B}'$ can be enlarged to an independent set of nodes V in $\mathcal{B}$ by adding at most one node inside each one of the $d-1$ removed paths $v_i \rightsquigarrow H$. Conversely, if we remove from an independent subset V of $\mathcal{B}$ its nodes that are intermediate in the $d-1$ removed paths $v_i \rightsquigarrow H$ (and by the independence condition each such path will contain at most one element of V and therefore we are removing in this way at most d nodes from V), we obtain an independent subset V' of $\mathcal{B}'$. Then:

- If $H \notin \uparrow_{\mathcal{B},only}(\mathscr{E}_1)$, any maximal independent subset of $\uparrow_{\mathcal{B}',only}(\mathscr{E}_1)$ is also a maximal independent subset of $\uparrow_{\mathcal{B},only}(\mathscr{E}_1)$. Then, by Eqn. (16), for every maximal independent set $V \subseteq \uparrow_{\mathcal{B},only}(\mathscr{E}_1)$

$$|V| \leqslant \sum_{i=1}^{l_1} d_i + \sum_{i=l_1+1}^{l_1+k_1}(d_i - 1).$$

.

- If $H = H_{l_1+k_1} \in \uparrow_{\mathcal{B},only}(\mathscr{E}_1)$, any maximal independent subset of $\uparrow_{\mathcal{B}',only}(\mathscr{E}_1)$ can be enlarged to a maximal independent subset of $\uparrow_{\mathcal{B},only}(\mathscr{E}_1)$ by adding at most $d_{l_1+k_1} - 1$ nodes. Then, by Eqn. (16),

$$|V| \leqslant \sum_{i=1}^{l_1} d_i + \sum_{i=l_1+1}^{l_1+k_1-1}(d_i - 1) + (d_{l_1+k_1} - 1)$$

  for every maximal independent set $V \subseteq \uparrow_{\mathcal{B},only}(\mathscr{E}_1)$.

This finishes the proof of the inductive step. □

Returning to Lemma 9, if $\mathcal{B}$ is a semi-d-ary k-blob with l exit reticulations and $|\mathscr{E}_1| = l_1$, then each reticulation has in-degree at most d and $|\mathcal{I}_1| \leqslant k - l$, and then

$$|V| \leqslant \sum_{H_i \in \mathscr{E}_1} \deg_{in} H_i + \sum_{H_i \in \mathcal{I}_1} (deg_{in} H_i - 1) \leqslant |\mathscr{E}_1| \cdot d + (k - |\mathscr{E}|)(d-1)$$

as states Lemma 9.

2 Proof of Corollary 2

We first prove a refinement of Lemma 10 for level-1 networks.

Lemma 12. *Let $\mathcal{B}$ be a semi-d-ary level-1 blob with exit reticulation H and let X, X' be two multisets of nodes of $\mathcal{B}$ with $|X'| < |X|$ satisfying the following two further conditions:*

(i) For each $v \in V(\mathcal{B})$, if $m_{X'}(v) < m_X(v)$, then $m_X(v) = 1$ and $m_{X'}(v) = 0$.
(ii) $H \in X \cup X'$.

Then

$$\uparrow X \cap \uparrow X' \subseteq \uparrow \tau_{A,B}(X) \cap \uparrow \tau_{B,A}(X') \tag{17}$$

for some subsets $A \subseteq \operatorname{Supp} X \setminus \operatorname{Supp} X'$ and $B \subseteq \{H\} \cap (\operatorname{Supp} X' \setminus \operatorname{Supp} X)$ such that either $B = \emptyset$ and $|A| = 1$, or $B = \{H\}$ and $1 < |A| \leqslant d$.

Proof. Using the same notations as in the proof of Lemma 10, observe that the Eqn. (6) therein holds identically in this case, i.e.,

$$0 < |X| - |X'| \leqslant |\widehat{X}| - |\widehat{X}'|. \tag{18}$$

In addition, $\mathscr{E} = \{H\}$. Now consider the following cases:

(a) If there exists some $x \in \widehat{X}$ with a proper descendant in X, then $A = \{x\}$, $B = \emptyset$ satisfy the required properties as proved in case **(a)** of Lemma 10.

(b) If $H \in X$ and no $x \in \widehat{X}$ has any proper descendant in X, then $\widehat{X} = \{H\}$, and then $\widehat{X}' = \emptyset$ by Eqn. (18) and $\uparrow X' = \uparrow(X \setminus \{H\})$ because, since $\widehat{X}' = \emptyset$, $X' = X \setminus \widehat{X} = X \setminus \{H\}$. Then, $A = \{H\}$ and $B = \emptyset$ satisfy the required properties.

(c) If $H \notin X$ and no $x \in \widehat{X}$ has any proper descendant in X, then, on the one hand, H belongs to $\operatorname{Supp} X' \setminus \operatorname{Supp} X$ by condition *(ii)*, and hence $H \in \widehat{X}'$, and, on the other hand, $\widehat{X}$ is an independent set of at most d nodes (because there are at most d different paths from the root to H).

For brevity, let X'' denote the full sub-multiset of X' supported on $\operatorname{Supp} X' \setminus \{H\}$. By Eqn. (18),

$$|\widehat{X}| > |\widehat{X}'| \geqslant |\operatorname{Supp} X'| = |\operatorname{Supp} X''| + 1$$

and thus $|\widehat{X}| \geqslant |\operatorname{Supp} X''| + 2$. Now, since $\mathcal{B}$ does not contain internal reticulations and the nodes in $\widehat{X}$ are independent, each node in X'' has at most one ancestor in $\widehat{X}$. This implies that $|\widehat{X} \cap \uparrow X''| \leqslant |\operatorname{Supp} X''|$ and hence

$$|\operatorname{Supp} X''| + 2 \leqslant |\widehat{X}| = |\widehat{X} \cap \uparrow X''| + |\widehat{X} \setminus \uparrow X''| \leqslant |\operatorname{Supp} X''| + |\widehat{X} \setminus \uparrow X''|,$$

which implies $|\widehat{X} \setminus \uparrow X''| \geqslant 2$.

Take then $A = \widehat{X} \setminus \uparrow X''$ and $B = \{H\}$. As we have just seen, $2 \leqslant |A| \leqslant |\widehat{X}| \leqslant d$. Thus, A, B satisfy the required properties in the statement. As far as Eqn. (17) goes, that is,

$$\uparrow X \cap \uparrow X' \subseteq \uparrow((X \setminus A) \cup \{H\}) \cap \uparrow((X' \setminus \{H\}) \cup A),$$

observe that, since H is the only exit reticulation of $\mathcal{B}$, $\uparrow H = V(\mathcal{B})$ and hence $\uparrow X' = \uparrow((X \setminus A) \cup \{H\}) = V(\mathcal{B})$. So, we actually must prove that

$$\uparrow X \subseteq \uparrow(X' \setminus \{H\}) \cup \uparrow A.$$

We must consider two cases.

- If $m_{X'}(H) > 1$, then $H \in X' \setminus \{H\}$ and thus $\uparrow(X' \setminus \{H\}) = V(\mathcal{B})$ and the desired inclusion is obvious.
- If $m_{X'}(H) = 1$, then $X' \setminus \{H\} = X''$, and in this case

$$\begin{aligned}\uparrow X &= \uparrow\widehat{X} \cup \uparrow(X \setminus \widehat{X}) = \uparrow(\widehat{X} \cap \uparrow X'') \cup \uparrow(\widehat{X} \setminus \uparrow X'') \cup \uparrow(X' \setminus \widehat{X}') \\ &\subseteq \uparrow X'' \cup \uparrow A \cup \uparrow(X' \setminus \{H\}) = \uparrow(X' \setminus \{H\}) \cup \uparrow A\end{aligned}$$

as desired.

This finishes the proof of case **(c)**. □

We can proceed now with the proof of Corollary 2. Arguing as in the proof of Theorem 1, we can assume that N is at-most-bifurcating and in particular that no node in N is the split node of more than one blob. Notice moreover that now each blob has only one reticulation: its exit reticulation.

We follow the proof of Theorem 1 by induction on the number α of arcs of the network. The base case $\alpha = 0$ is again obvious, and thus we must only consider the inductive step. So, let N be a semi-d-ary level-1 phylogenetic network on Σ with more than one arc, and let $X, X' \subseteq \Sigma$ with $|X'| < |X|$. If $|X| = 1$ the exchange property is trivially satisfied taking $(A, B) = (X, \emptyset) \in \mathscr{S}_0$, so we assume $|X| \geqslant 2$.

Arguing as in cases **(a)**, **(b)** and **(c.1)** in the proof of Theorem 1, we can assume that:

(i) *The root r is the split node of a single, semi-d-ary blob $\mathcal{B}$.*
(ii) *For every node v in $\mathcal{B}$, if v has a child $\overline{v}$ outside $\mathcal{B}$ such that $|X \cap C(\overline{v})| > |X' \cap C(\overline{v})|$, then $|X \cap C(\overline{v})| = 1$ and $|X' \cap C(\overline{v})| = 0$.*
(iii) *$C(H) \cap (X \cup X') \neq \emptyset$.*

We use henceforth the same notations as in point **(c.2)** in the proof of that theorem. The hypotheses of Lemma 12 are satisfied by $\mathcal{B}^*_X$ and $\mathcal{B}^*_{X'}$. Then, there exist two sets $\mathcal{B}_A, \mathcal{B}_B \subseteq V(\mathcal{B})$ such that $\mathcal{B}_A \subseteq \operatorname{Supp} \mathcal{B}^*_X \setminus \operatorname{Supp} \mathcal{B}^*_{X'}$, $\mathcal{B}_B \subseteq \{H\} \cap (\operatorname{Supp} \mathcal{B}^*_{X'} \setminus \operatorname{Supp} \mathcal{B}^*_X)$, $|\mathcal{B}_B| = 0$ and $|\mathcal{B}_A| = 1$, or $|\mathcal{B}_B| = 1 < |\mathcal{B}_A| \leqslant d$, and

$$\uparrow\mathcal{B}^*_X \cap \uparrow\mathcal{B}^*_{X'} \subseteq \uparrow\tau_{\mathcal{B}_A,\mathcal{B}_B}(\mathcal{B}^*_X) \cap \uparrow\tau_{\mathcal{B}_B,\mathcal{B}_A}(\mathcal{B}^*_{X'}). \tag{19}$$

Now take $A = \bigcup_{v \in \mathcal{B}_A} (X \cap C(\overline{v}))$ and, if $\mathcal{B}_B = \{H\}$, choose any $b \in X' \cap C(H)$ and take $B = \{b\}$, while if $\mathcal{B}_B = \emptyset$, take $B = \emptyset$. Notice that if $B = \emptyset$, then $|\mathcal{B}_A| = 1$ and hence $|A| = 1$, while, if $|B| = 1$, then $H \in \operatorname{Supp} \mathcal{B}^*_{X'} \setminus \operatorname{Supp} \mathcal{B}^*_X$ and hence $X \cap C(H) = \emptyset$.

These sets satisfy $|A| = |\mathcal{B}_A|$, $|B| = |\mathcal{B}_B|$ and thus $(A, B) \in \mathscr{S}_d$. Now, arguing as in the proof of Theorem 1, we have $\mathcal{B}_A = \mathcal{B}^*_A$, $\mathcal{B}_B = \mathcal{B}^*_B$, and

$$\tau_{\mathcal{B}_A,\mathcal{B}_B}(\mathcal{B}^*_X) = \mathcal{B}^*_{\tau_{A,B}(X)} \text{ and } \operatorname{Supp} \mathcal{B}^*_{\tau_{A,B}(X)} = ((\operatorname{Supp} \mathcal{B}^*_X) \setminus \mathcal{B}_A) \cup \mathcal{B}_B, \tag{20}$$

$$\begin{aligned}\tau_{\mathcal{B}_B,\mathcal{B}_A}(\mathcal{B}^*_{X'}) = \mathcal{B}^*_{\tau_{B,A}(X')} \text{ and } \operatorname{Supp} \mathcal{B}^*_{\tau_{B,A}(X')} &= \operatorname{Supp} \mathcal{B}_{X' \setminus B} \cup \mathcal{B}_A \\ &\supseteq ((\operatorname{Supp} \mathcal{B}^*_{X'}) \setminus \mathcal{B}_B) \cup \mathcal{B}_A\end{aligned} \tag{21}$$

(where the inclusion in (21) is not an equality if $\mathcal{B}_B = \{H\}$ and $m_{\mathcal{B}^*_{X'}}(H) = |X' \cap C(H)| > 1 = m_{\mathcal{B}_B}(H)$) and, still arguing as in the aforementioned proof,

$$\begin{aligned}&\mathrm{rPSD}_N(X) - \mathrm{rPSD}_N(\tau_{A,B}(X)) =\\ &= \sum_{v\in\mathcal{B}^*_A} \mathrm{rPSD}_{\overline{N}_v}(A) - \sum_{v\in\mathcal{B}^*_B} \mathrm{rPSD}_{\overline{N}_v}(B) + \sum_{e\in\uparrow\mathcal{B}^*_X} w(e) - \sum_{e\in\uparrow\mathcal{B}^*_{\tau_{A,B}(X)}} w(e).\end{aligned}$$

Now, the difference between Eqn. (21) above and Eqn. (12) in the proof of Theorem 1 makes dealing with $\mathrm{rPSD}_N(\tau_{B,A}(X')) - \mathrm{rPSD}_N(X')$ different here from in the aforementioned proof. In the current situation, we have that

$$\begin{aligned}&\sum_{v\in\mathrm{Supp}\,\mathcal{B}^*_{\tau_{B,A}(X')}} \mathrm{rPSD}_{\overline{N}_v}\big(\tau_{B,A}(X')\big) =\\ &\qquad= \sum_{v\in\mathrm{Supp}\,\mathcal{B}^*_{X'\setminus B}} \mathrm{rPSD}_{\overline{N}_v}\big((X'\setminus B)\cup A\big) + \sum_{v\in\mathcal{B}_A} \mathrm{rPSD}_{\overline{N}_v}\big((X'\setminus B)\cup A\big)\\ &\qquad= \sum_{v\in\mathrm{Supp}\,\mathcal{B}^*_{X'\setminus B}} \mathrm{rPSD}_{\overline{N}_v}(X'\setminus B) + \sum_{v\in\mathcal{B}_A} \mathrm{rPSD}_{\overline{N}_v}(A)\end{aligned}$$

(because, if $v \in \mathcal{B}^*_{X'\setminus B}$, then $A \cap C(\bar{v}) = \emptyset$, and if $v \in \mathcal{B}_A$, then $X' \cap C(\bar{v}) = \emptyset$);

$$\begin{aligned}&\sum_{v\in\mathrm{Supp}\,\mathcal{B}^*_{X'}} \mathrm{rPSD}_{\overline{N}_v}(X') = \sum_{v\in(\mathrm{Supp}\,\mathcal{B}^*_{X'})\setminus\mathcal{B}_B} \mathrm{rPSD}_{\overline{N}_v}(X') + \sum_{v\in\mathcal{B}_B} \mathrm{rPSD}_{\overline{N}_v}(X')\\ &\leqslant \sum_{v\in(\mathrm{Supp}\,\mathcal{B}^*_{X'})\setminus\mathcal{B}_B} \mathrm{rPSD}_{\overline{N}_v}(X') + \sum_{v\in\mathcal{B}_B} \mathrm{rPSD}_{\overline{N}_v}(X'\setminus B) + \sum_{v\in\mathcal{B}_B} \mathrm{rPSD}_{\overline{N}_v}(B)\\ &\text{(by the subadditivity of rPSD)}\\ &= \sum_{v\in(\mathrm{Supp}\,\mathcal{B}^*_{X'})\setminus\mathcal{B}_B} \mathrm{rPSD}_{\overline{N}_v}(X'\setminus B) + \sum_{v\in\mathcal{B}_B} \mathrm{rPSD}_{\overline{N}_v}(X'\setminus B) + \sum_{v\in\mathcal{B}_B} \mathrm{rPSD}_{\overline{N}_v}(B)\\ &\text{(because, } B\cap C(\bar{v}) = \emptyset \text{ if } v\notin\mathcal{B}_B)\\ &= \sum_{v\in\mathrm{Supp}\,\mathcal{B}^*_{X'\setminus B}} \mathrm{rPSD}_{\overline{N}_v}(X'\setminus B) + \sum_{v\in\mathcal{B}_B} \mathrm{rPSD}_{\overline{N}_v}(B);\end{aligned}$$

and then

$$\begin{aligned}&\mathrm{rPSD}_N(\tau_{B,A}(X')) - \mathrm{rPSD}_N(X')\\ &\quad= \sum_{v\in\mathrm{Supp}\,\mathcal{B}^*_{\tau_{B,A}(X')}} \mathrm{rPSD}_{\overline{N}_v}(\tau_{B,A}(X')) - \sum_{v\in\mathrm{Supp}\,\mathcal{B}^*_{X'}} \mathrm{rPSD}_{\overline{N}_v}(X') + \sum_{e\in\uparrow\mathcal{B}^*_{\tau_{B,A}(X')}} w(e) - \sum_{e\in\uparrow\mathcal{B}^*_{X'}} w(e)\\ &\quad\geqslant \sum_{v\in\mathrm{Supp}\,\mathcal{B}^*_{X'\setminus B}} \mathrm{rPSD}_{\overline{N}_v}(X'\setminus B) + \sum_{v\in\mathcal{B}_A} \mathrm{rPSD}_{\overline{N}_v}(A)\end{aligned}$$

$$- \sum_{v \in \operatorname{Supp} \mathcal{B}^*_{X' \setminus B}} \mathrm{rPSD}_{\overline{N}_v}(X' \setminus B) - \sum_{v \in \mathcal{B}_B} \mathrm{rPSD}_{\overline{N}_v}(B) + + \sum_{e \in \uparrow \mathcal{B}^*_{\tau_{B,A}(X')}} w(e) - \sum_{e \in \uparrow \mathcal{B}^*_{X'}} w(e).$$

$$= \sum_{v \in \mathcal{B}_A} \mathrm{rPSD}_{\overline{N}_v}(A) - \sum_{v \in \mathcal{B}_B} \mathrm{rPSD}_{\overline{N}_v}(B) + \sum_{e \in \uparrow \mathcal{B}^*_{\tau_{B,A}(X')}} w(e) - \sum_{e \in \uparrow \mathcal{B}^*_{X'}} w(e).$$

Then, as in Theorem 1, we conclude that

$$\mathrm{rPSD}_N(X) - \mathrm{rPSD}_N(\tau_{A,B}(X)) \leqslant \mathrm{rPSD}_N(\tau_{B,A}(X')) - \mathrm{rPSD}_N(X')$$

if

$$\sum_{e \in \uparrow \mathcal{B}^*_X} w(e) - \sum_{e \in \uparrow \mathcal{B}^*_{\tau_{A,B}(X)}} w(e) \leqslant \sum_{e \in \uparrow \mathcal{B}^*_{\tau_{B,A}(X')}} w(e) - \sum_{e \in \uparrow \mathcal{B}^*_{X'}} w(e),$$

and this last inequality is deduced from Eqn. (19) as in the proof of Theorem 1.

3 Proof of Proposition 8

To begin with, notice that

$$\mathscr{S}_{2,3} = \mathscr{S}_0 \cup \{(A,B) \in \mathcal{P}(\Sigma)^2 : 1 \leqslant |B| < |A| < 6,\ |A| - |B| \leqslant 4\},$$
$$\mathscr{S}_{1,5} = \mathscr{S}_0 \cup \{(A,B) \in \mathcal{P}(\Sigma)^2 : 1 \leqslant |B| < |A| \leqslant 5\}$$

and therefore $\mathscr{S}_{1,5} = \mathscr{S}_{2,3}$. To simplify the notation, we shall abbreviate Opt-$\tau_{1,5,j}$ = Opt-$\tau_{2,3,j}$ by simply Opt-τ_j. Observe that in both cases considered in the statement j can go from 1 to 4.

Let Y be an optimal sequence of N and fix $1 < m \leqslant n$. To ease the task of the reader, we sketch the flow of the proof in Figure 10.

By Theorem 1,

$$(m, m-1) \prec^Y (m - j_1,\ m - 1 + j_1) \tag{22}$$

for some $j_1 \in \{1, 2, 3, 4\}$.

(1) If $j_1 = 1$, then, we conclude as in (1) in the proof of Proposition 6 that $Y_m \in$ Opt-$\tau_1(\mathrm{Opt}_{m-1})$ and $Y_{m-1} \in$ Opt-$\tau_1^{-1}(\mathrm{Opt}_m)$.

(2) If $j_1 = 2$, then $(m - j_1, m - 1 + j_1) = (m - 2, m + 1)$. Applying Theorem 1 again,

$$(m+1, m-2) \prec^Y (m + 1 - j_2, m - 2 + j_2),$$

for some $j_2 \in \{1, 2, 3, 4\}$.

(2.a) If $j_2 = 1$ or $j_2 = 2$, we conclude as in (2) in the proof of Proposition 6 that $Y_m \in$ Opt-$\tau_2(\mathrm{Opt}_{m-2})$ and $Y_{m-1} \in$ Opt-$\tau_2^{-1}(\mathrm{Opt}_{m+1})$.

(2.b) When $j_2 = 3$, we have $(m+1, m-2) \prec^Y (m-2, m+1)$ and, as in (2.b) in the proof of Proposition 6, we can only conclude that $Y_{m+1} \in$ Opt-$\tau_3(\mathrm{Opt}_{m-2})$ and $Y_{m-2} \in$ Opt-$\tau_3^{-1}(\mathrm{Opt}_{m+1})$.

$$(m,m-1)\begin{cases}
\prec_1^Y (m-1,m) \Rightarrow \text{(a)}\\
\prec_2^Y \{m+1,m-2\}\begin{cases} \prec_{1,2}^Y \{m,m-1\} \Rightarrow \text{(a)}\\ \prec_3^Y\{m-2,m+1\} \Rightarrow \text{(b)}\\ \prec_4^Y\{m-3,m+2\}\begin{cases}\prec_{1,4}^Y\{m+1,m-2\}\Rightarrow\text{(b)}\\ \prec_{2,3}^Y\{m,m-1\}\Rightarrow\text{(a)}\end{cases}\end{cases}\\
\prec_3^Y\{m+2,m-3\}\begin{cases}\prec_1^Y(m+1,m-2)\begin{cases}\prec_{1,2}^Y\{m,m-1\}\Rightarrow\text{(a)}\\ \prec_3^Y\{m-2,m+1\}\Rightarrow\text{(b)}\\ \prec_4^Y\{m-3,m+2\}\begin{cases}\prec_{1,4}^Y\{m+1,m-2\}\Rightarrow\text{(b)}\\ \prec_{2,3}^Y\{m,m-1\}\Rightarrow\text{(a)}\end{cases}\end{cases}\\ \prec_{2,3}^Y\{m,m-1\}\Rightarrow\text{(a)}\\ \prec_4^Y\{m-2,m+1\}\begin{cases}\prec_{1,2}^Y\{m,m-1\}\Rightarrow\text{(a)}\\ \prec_3^Y\{m-2,m+1\}\Rightarrow\text{(b)}\\ \prec_4^Y\{m-3,m+2\}\Rightarrow \text{(a) or (b) as after } (m+2,m-3)\prec_1^Y\{m+1,m-2\}\end{cases}\end{cases}\\
\prec_4^Y\{m-4,m+3\}\begin{cases}\prec_{1,2}^Y\{m+2,m-3\}\Rightarrow \text{(a) or (b) as after } (m,m-1)\prec_3^Y\{m+2,m-3\}\\ \prec_{3,4}^Y\{m,m-1\}\Rightarrow\text{(a)}\end{cases}
\end{cases}$$

Fig. 10 Sketch of the proof of Proposition 8. To make the diagram shorter, we write $(p,q) \prec^Y_{j_1,j_2} \{p',q'\}$ to mean that $(p,q) \prec^Y_{j_1} \{p',q'\}$ or $(p,q) \prec^Y_{j_2} \{p',q'\}$.

(2.c) When $j_2 = 4$, we have $(m+1,m-2) \prec^Y (m-3,m+2)$. Applying Theorem 1 again,

$$(m+2,m-3) \prec^Y (m+2-j_3, m-3+j_3),$$

for some $j_3 \in \{1,2,3,4\}$. Now:

(2.c.i) If $j_3 = 1$ or 4, $\{m+2-j_3, m-3+j_3\} = \{m+1, m-2\}$ and, as in (2.b), we conclude that $Y_{m+1} \in \text{Opt-}\tau_3(\text{Opt}_{m-2})$ and $Y_{m-2} \in \text{Opt-}\tau_3^{-1}(\text{Opt}_{m+1})$.

(2.c.ii) If $j_3 = 2$ or 3, $\{m+2-j_3, m-3+j_3\} = \{m, m-1\}$ and, as in (2.a), we conclude that $Y_m \in \text{Opt-}\tau_2(\text{Opt}_{m-2})$ and $Y_{m-1} \in \text{Opt-}\tau_2^{-1}(\text{Opt}_{m+1})$.

(3) If $j_1 = 3$, then $(m-j_1, m-1+j_1) = (m-3, m+2)$. Applying Theorem 1 again,

$$(m+2,m-3) \prec^Y (m+2-j_2, m-3+j_2),$$

for some $j_2 \in \{1,2,3,4\}$.

(3.a) If $j_2 = 2$ or 3, $\{m+2-j_2, m-3+j_2\} = \{m, m-1\}$, closing the $\prec$-chain initiated with (22). Then, by Corollary 5, $Y_m \in \text{Opt-}\tau_3(\text{Opt}_{m-3})$ and $Y_{m-1} \in \text{Opt-}\tau_3^{-1}(\text{Opt}_{m+2})$.

(3.b) If $j_2 = 1$ or 4, $\{m+2-j_2, m-3+j_2\} = \{m+1, m-2\}$. But now we can follow as in case (2) and we conclude that one of the following situations must hold:

- $Y_m \in \text{Opt-}\tau_3(\text{Opt}_{m-3})$ and $Y_{m-1} \in \text{Opt-}\tau_3^{-1}(\text{Opt}_{m+2})$,
- $Y_{m+1} \in \text{Opt-}\tau_3(\text{Opt}_{m-2})$ and $Y_{m-2} \in \text{Opt-}\tau_3^{-1}(\text{Opt}_{m+1})$.

(4) If $j_1 = 4$, then $(m-j_1, m-1+j_1) = (m-4, m+3)$. Applying Theorem 1 again,

$$(m+3,m-4) \prec^Y (m+3-j_2, m-4+j_2),$$

for some $j_2 \in \{1,2,3,4\}$.

(4.a) If $j_2 = 3$ or 4, $\{m+3-j_2, m-4+j_2\} = \{m, m-1\}$, closing the $\prec$-chain initiated with (22). Then, by Corollary 5, $Y_m \in \text{Opt-}\tau_4(\text{Opt}_{m-4})$ and $Y_{m-1} \in \text{Opt-}\tau_4^{-1}(\text{Opt}_{m+3})$.

(4.b) If $j_2 = 1$, $(m+3, m-4) \prec^Y (m+2, m-3)$ and we can follow as in (3), obtaining that one of the following situations must hold:
- $Y_m \in \text{Opt-}\tau_4(\text{Opt}_{m-4})$ and $Y_{m-1} \in \text{Opt-}\tau_4^{-1}(\text{Opt}_{m+3})$,
- $Y_{m+1} \in \text{Opt-}\tau_3(\text{Opt}_{m-2})$ and $Y_{m-2} \in \text{Opt-}\tau_3^{-1}(\text{Opt}_{m+1})$.

(4.c) If $j_2 = 2$, $(m+3, m-4) \prec^Y (m+1, m-2)$ and we can follow as in (2), obtaining that one of the following situations must hold:
- $Y_m \in \text{Opt-}\tau_4(\text{Opt}_{m-4})$ and $Y_{m-1} \in \text{Opt-}\tau_4^{-1}(\text{Opt}_{m+3})$,
- $Y_{m+1} \in \text{Opt-}\tau_3(\text{Opt}_{m-2})$ and $Y_{m-2} \in \text{Opt-}\tau_3^{-1}(\text{Opt}_{m+1})$.

Summarizing, we have two possibilities: either

$$Y_m \in \bigcup_{j=1}^{4} \text{Opt-}\tau_j(\text{Opt}_{m-j}) \text{ and } Y_{m-1} \in \bigcup_{j=1}^{4} \text{Opt-}\tau_j^{-1}(\text{Opt}_{m-1+j})$$

or

$$Y_{m+1} \in \text{Opt-}\tau_3(\text{Opt}_{m-2}) \text{ and } \text{Opt-}\tau_3(Y_{m-2}) \subseteq \text{Opt}_{m+1}.$$

By the arbitrary choice of Y and m, this concludes the proof.

4 Some examples

Example 4. *Consider the phylogenetic networks in Figure 11: above, a semi-4-ary level-1 network and below, a semibinary level-3 network.*

In both cases we have the following optimal sets of leaves:

$$\begin{aligned}
\text{Opt}_0 &: \emptyset \\
\text{Opt}_1 &: \{z_0\} \\
\text{Opt}_2 &: \{z_0, z_1\} \\
\text{Opt}_3 &: \{x_{12}, x_{13}, z_0\} \\
\text{Opt}_4 &: \{x_{11}, x_{12}, x_{13}, z_0\} \\
\text{Opt}_5 &: \{x_{00}, x_{01}, x_{02}, x_{03}, z_1\} \\
\text{Opt}_6 &: \{x_{00}, x_{01}, x_{02}, x_{03}, x_{12}, x_{13}\} \\
\text{Opt}_7 &: \{x_{00}, x_{01}, x_{02}, x_{03}, x_{11}, x_{12}, x_{13}\} \\
\text{Opt}_8 &: \{x_{00}, x_{01}, x_{02}, x_{03}, x_{10}, x_{11}, x_{12}, x_{13}\} \\
\text{Opt}_9 &: \{x_{00}, x_{01}, x_{02}, x_{03}, x_{10}, x_{11}, x_{12}, x_{13}, z_1\} \\
\text{Opt}_{10} &: \{x_{00}, x_{01}, x_{02}, x_{03}, x_{10}, x_{11}, x_{12}, x_{13}, z_0, z_1\}
\end{aligned}$$

In the case of the semi-4-ary level-1 network we have

$$\text{Opt}_5 \not\subseteq \text{Opt-}\tau_1(\text{Opt}_4) \cup \text{Opt-}\tau_2(\text{Opt}_3) = \big\{\{x_{00}, x_{01}, x_{02}, x_{03}, x_{13}\}\big\}.$$

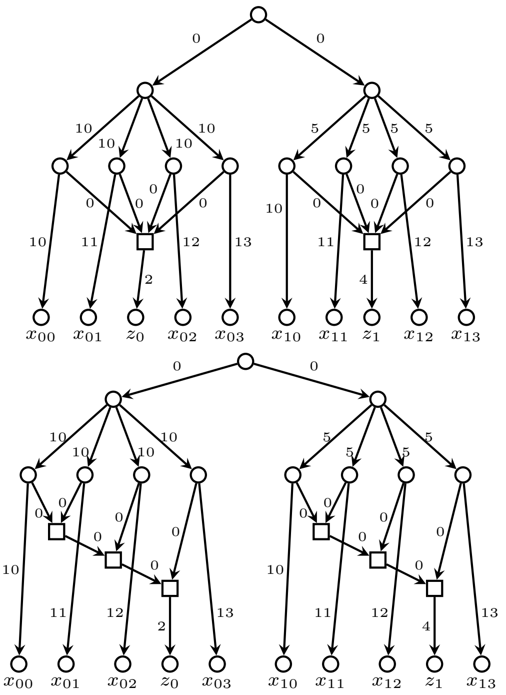

Fig. 11 The networks in Example 4.

However, for the level-3 network, $\mathrm{Opt}_m = \mathrm{Opt}\text{-}\tau_1(\mathrm{Opt}_{m-1})$ *and* $\mathrm{Opt}_m = \mathrm{Opt}\text{-}\tau_1^{-1}(\mathrm{Opt}_{m+1})$ *for all* $1 \leqslant m \leqslant n = 10$ *and* $0 \leqslant m < n$ *respectively. This should not be surprising, because in Example 11 we showed that for this network (although with different weights) we can always find an* rPSD*-improving pair* (A, B) *with* $|A| - |B| = 1$*, hence the first case in the proof of Proposition 7 could always be chosen and prove that* $\mathrm{Opt}_m \subseteq \mathrm{Opt}\text{-}\tau_1(\mathrm{Opt}_{m-1})$*.*

As we mention in Example 4, if some network has some Opt_m not included into $\bigcup_{j=1}^{3} \mathrm{Opt}\text{-}\tau_{k,d,j}(\mathrm{Opt}_{m-j})$, then that network has two sets of leaves X, X' with $m = |X| = |X'| + 1$ and no rPSD-improving pairs (A, B) with $|A| - |B| < 3$. Otherwise, if we could always find some rPSD-improving pair with $|A| - |B| < 3$, the proof of Propositions 7 and 8 would never need to explore the cases $j_1 \in \{3, 4\}$ and thus obtain a similar result to Proposition 6. In Example 5 we show a semi-5-ary network that has $X = \{x_{00}, \ldots, x_{04}, z_1\}$ and $X' = \{x_{10}, \ldots, x_{13}, z_0\}$ with only rPSD-improving pairs with $|A| - |B| \geqslant 3$, yet the obvious greedy algorithm would still work in this network. In contrast, we have not found any semibinary level-3 network that has some $X, X' \subseteq \Sigma$ with $|X| = |X'| + 1$ and no rPSD-improving pair (A, B) with $|A| - |B| = 1$.

Example 5. *The semi-5-ary level-1 network in Figure 12, analogous to the semi-4-ary network from Example 4, similarly has* $\{x_{00}, \ldots, x_{04}, z_1\} \in \mathrm{Opt}_6 \setminus \bigcup_{j=1}^{3} \mathrm{Opt}\text{-}\tau_j(\mathrm{Opt}_{6-j})$ *but still, for all* $1 \leqslant m \leqslant n$*,* $\mathrm{Opt}_m \subseteq \bigcup_{j=1}^{4} \mathrm{Opt}\text{-}\tau_j(\mathrm{Opt}_{m-j})$*.*

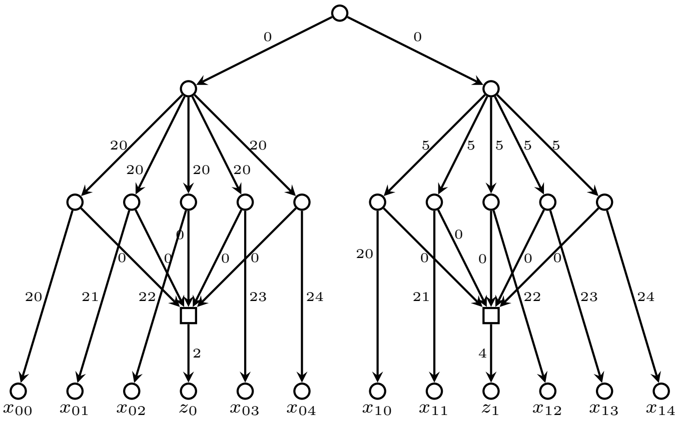

Fig. 12 The network in Example 5.
